# Amidase‐Catalyzed Desorption of CO_2_ Captured in Aqueous Monoethanolamine (MEA) Solutions

**DOI:** 10.1002/anie.5406475

**Published:** 2026-06-26

**Authors:** Yang Yang, Onur Kırtel, Silke Flindt Badino, Lea Helena Strother, Laura Rotilio, Jerik Mathew Valera Lauridsen, Stefanie Neun, Jens Preben Morth, Ji‐Woong Lee, Ditte Hededam Welner, Peter Westh

**Affiliations:** ^1^ Protein and Enzyme Technology Department of Biotechnology and Biomedicine Technical University of Denmark Lyngby Denmark; ^2^ The Novo Nordisk Foundation CO_2_ Research Center (CORC) Aarhus University Aarhus Denmark; ^3^ The Novo Nordisk Foundation Biotechnology Research Institute For the Green Transition Technical University of Denmark Lyngby Denmark; ^4^ Department of Chemistry University of Copenhagen Copenhagen Denmark; ^5^ Novonesis Lyngby Denmark

**Keywords:** amidase/urethanase, biocatalysis, carbon capture, CO_2_ desorption, monoethanolamine

## Abstract

Aqueous monoethanolamine (MEA) solutions can absorb CO_2_ from industrial point sources via amine scrubbing. Mechanistically, CO_2_ diffuses in and reacts with MEA or hydroxide to form a mixture of carbamate and carbonate/bicarbonate. Subsequently, CO_2_ is released for storage or utilization via an energy‐intensive thermal solvent‐regeneration process. Here, we explored biocatalytic acceleration of the solvent regeneration step, which accounts for the dominant energy cost of carbon dioxide removal (CDR) processes. We conducted a sequence mining campaign based on urethane‐degrading Amidase Signature superfamily enzymes and discovered amidases that hydrolyze MEA carbamate, thereby increasing the overall release rate of CO_2_. The most promising candidate, an amidase from *Parageobacillus caldoxylosilyticus* (PcAmd), showed good thermostability (*T*_m_ around 70°C) and a specific activity against MEA carbamate of about 1 U/mg (20 nKat/mg). We found that PcAmd accelerated the regeneration of MEA sorbent. Specifically, PcAmd at 1 µM increased the initial CO_2_ release rate by about 20%, and the time required to release 80% of the captured CO_2_ was reduced by approximately half compared to enzyme‐free solutions. These results identified a novel potential of amidases in carbon capture and motivated further efforts to discover or engineer enzymes with better stability and activity for industrial CDR applications.

## Graphical abstract

CO_2_ is captured in aqueous monoethanolamine (MEA) sorbents as carbamate and bicarbonate/carbonate. The subsequent desorption for storage or utilization is energy‐intensive, and this is a major impediment to industrial carbon dioxide removal (CDR) processes. The desorption process can be promoted by enzymes that catalyze the dissociation of distinct CO_2_‐bound species, as illustrated in the figure, thereby accelerating solvent regeneration.

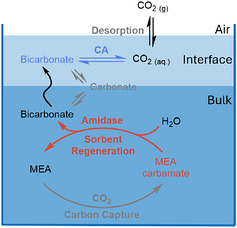

Developing efficient and sustainable carbon capture (CC) technologies is essential to reducing greenhouse gas emissions. One key approach is chemical CC, which uses chemical reactions to separate CO_2_ from gas streams. Chemical CC has been studied for a range of alkaline sorbents, [1, 2, 3, 4] and aqueous amines have appeared as particularly promising candidates [5, 6]. Currently, the industrial standard for CO_2_ scrubbing is a 20%‒30% w/w aqueous solution of monoethanolamine (MEA) [7, 8]. This sorbent is favored by rapid kinetics, but shows shortcomings such as chemical instability, oxidative and evaporative losses, environmental impacts of evaporation, and a high overall energy consumption [5, 9, 10, 11, 12]. These issues are primarily rooted in the need for high temperatures to regenerate MEA solvents (so‐called CO_2_ stripping), partly because of the increased stability of MEA carbamate when the media becomes more basic during CO_2_ desorption [13, 14]. Typically, MEA regeneration involves heating to about 120°C, and energy costs for this step account for 60%–80% of operating expenses in MEA‐based CO_2_ scrubbing [15]. The need for high regeneration temperatures is driven by both thermodynamic and kinetic constraints, and the latter could be lessened by catalyzed desorption. It follows that catalyzed CO_2_ stripping has significant potential to reduce the energy intensity of CDR and, hence, the cost of the process. Inorganic metal‐based catalysts have been investigated for this purpose [16, 17], even though the mechanism remained unclear, mainly due to the complex nature of the amine‐CO_2_‐water system [13, 14].

The idea of enzymatic cleavage of the alkanolamine‐carbamate bonds for efficient solvent regeneration was put forward in a review by Salmon and House, [18] but to the best of our knowledge, it has never been pursued experimentally. In the current work, we did so with a focus on amidases (EC 3.5.1.x), a broad class of enzymes that hydrolyze amide bonds in a wide variety of compounds. We speculate that enzymatic hydrolysis of the MEA carbamate could accelerate the overall solvent regeneration process via the mechanism illustrated in Scheme 1 and Figure 4B.

**SCHEME 1:**
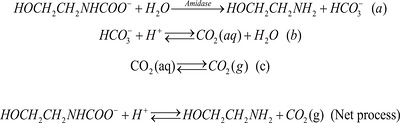
Suggested mechanism for enzyme‐catalyzed release of CO_2_ from MEA carbamate. Step (*a*) describes the amidase‐catalyzed hydrolysis of carbamate. We emphasize that PcAmd catalyzes only this step. The hydrolytic step is followed by dehydration of bicarbonate (*b*), and release of CO_2_ to the gas phase (*c*). For ease of reading, this scheme ignores the different protonation states of MEA and (bi)carbonate (see main text for details) in the full picture of a more complex reaction networks [13, 14].

To discover relevant enzymes, we concentrated on the Amidase Signature (AS) superfamily, which comprises the largest group of amidases, with over 133,000 sequences identified as of December 2025 (https://www.ebi.ac.uk/interpro/). Enzymes in this family are evolutionarily related to aspartic proteinases [19] and may hydrolyze both aromatic, aliphatic amides as well as α‐hydroxyamides [20]. Some amidases can also hydrolyze carbamate (or urethane) bonds. A subset of this group is considered to have carbamates as their native substrate and is hence termed urethanases (EC 3.5.1.75) [21]. Amidases and urethanases have previously been considered for technical applications, including the breakdown of carbamate herbicides [22] and urethane, a potential carcinogen, in food and beverages [21]. More recently, amidases were suggested for chemo‐enzymatic recycling of the plastic polyurethane (PU) [23].

We conducted a sequence mining campaign based on four known urethane‐hydrolyzing amidases [23, 24] to discover potential enzymes capable of hydrolyzing MEA carbamate, spanning seven distinct clades of AS superfamily sequences from Bacillaceae, as shown in Figure 1. Out of twenty sequences chosen for recombinant expression (see Table S1), four enzymes from clade A and one enzyme from clade D in Figure 1 could be expressed in soluble form in *E. coli*.

**FIGURE 1:**
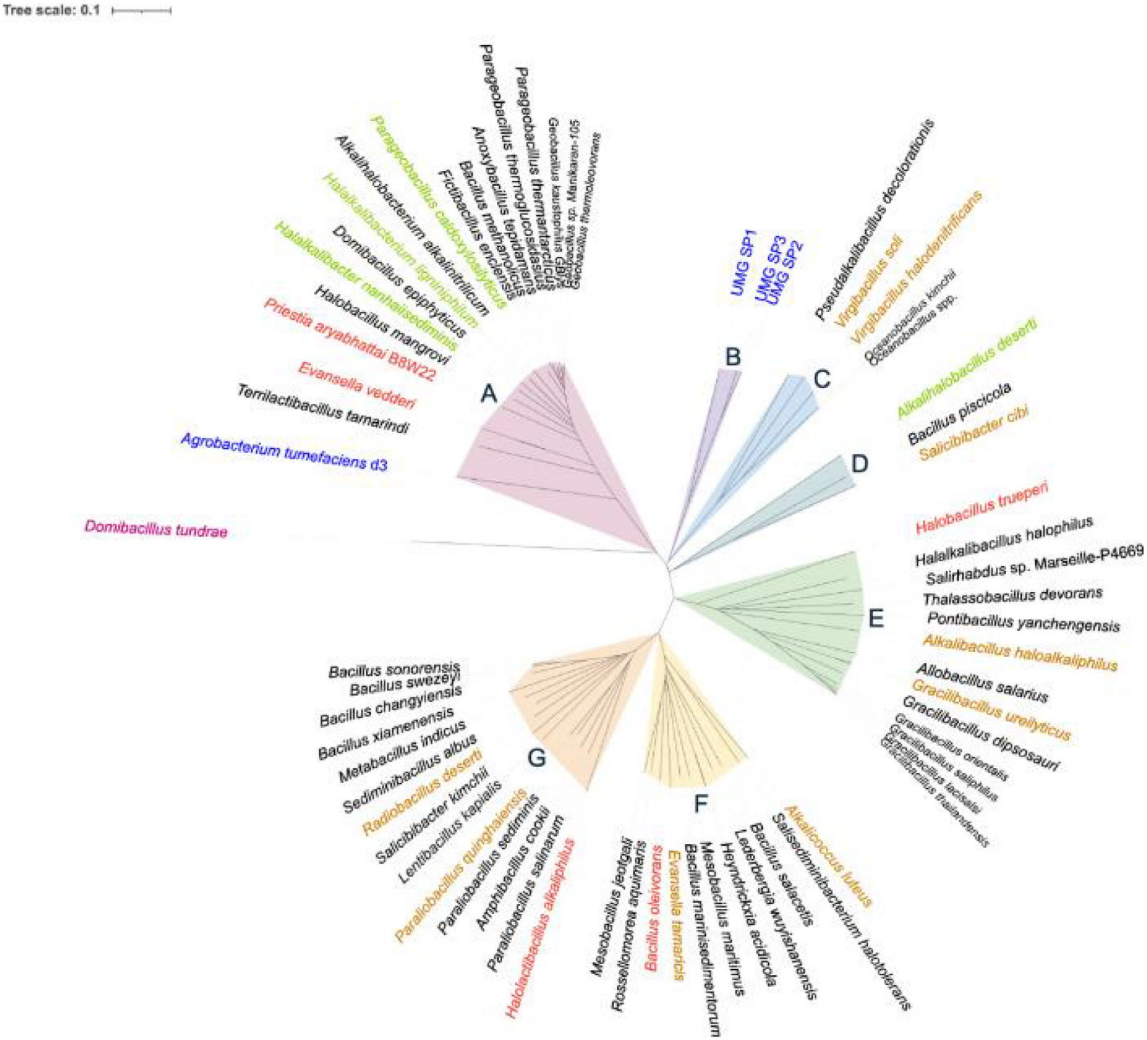
Phylogenetic tree showing the distribution of amidases investigated in this study. Names of the species are colored according to their recombinant expression and ability to accelerate desorption of CO_2_ bound in aqueous MEA (blue: query sequences, green: soluble & active, yellow: insoluble expression, red: no expression, purple: soluble & inactive (outgroup of the tree, not in a clade). At Amd is shown in blue since it is both a query sequence and active. Species names for clade B sequences are absent since they were discovered through metagenomic screening [23].

The release of CO_2_ from aqueous CO_2_/MEA solutions consumes protons, as shown in Scheme 1 and detailed in the (Supporting Information Section 6). Consequently, the desorption kinetics was monitored in a pH‐stat apparatus. Specifically, we first charged an aqueous MEA solution with CO_2_ using purge gas (10 vol% CO_2_/90 vol% N_2_). During this loading procedure, the pH was not kept constant but monitored as it decreased to the desired value (typically around pH 9.0 as specified for each experiment). Subsequently, at *t* = 0, we changed the purge gas to pure nitrogen and activated the pH‐stat titration system. The continuous removal of CO_2_(g) by the purged gas provided a driving force towards the right for the net reaction in Scheme 1, and the extent of acid titration, to maintain a constant pH, quantified the CO_2_ release. Initially, we screened all purified amidases for this study at pH 9.0 (Figure S6). Four amidases from clade A and one amidase from clade D were found to accelerate CO_2_ release while the enzyme from *Domibacillus tundrae* did not (Figures 1 and S6). The latter is annotated as an allophanate hydrolase on NCBI Conserved Domains, unlike the five active sequences, which were annotated simply as amidases [25, 26] Since we could not express and purify any soluble enzyme from clades C, E, F, and G, the potential of these clades remains unknown. Still, overall, the results suggested that clade A represented a promising group of sequences for biocatalytic MEA regeneration.

To further assess the applicability of the amidases, we tested their thermostability. Results in Figure S3A showed that most had moderate thermostability with transition temperatures in the 35°C‒55°C range at pH 7.6. The only exception was the amidase from *Parageobacillus caldoxylosilyticus* (PcAmd, native MW 55 kDa), which had a transition midpoint of about 72°C. PcAmd could be recombinantly expressed at high titers (ca. 50 mg protein per L of culture medium) in *E. coli*, and showed good CO_2_ release activity in the initial screens (Figure S6). PcAmd was also a promising candidate because it was only moderately destabilized at high pH (Figures S3B and S3C). The kinetic stability of PcAmd was tested (Figure S5), and we found half‐times for activity loss of about 2.2 h at 25°C and 40°C. At 60°C, the enzyme was severely destabilized, with a half‐life of only 0.4 h. In silico structural analysis of PcAmd (Figure S1) suggested that it formed a homodimer, and docking studies with MEA‐carbamate as ligand supported the view that this substrate may be hydrolyzed via the classical AS superfamily mechanism (Figure S2) [20, 27, 28, 29] These properties collectively suggested that PcAmd was the most promising candidate, and we focused on this enzyme in further work.

Before investigating enzyme catalysis, we used ^1^H NMR spectroscopy (Figure S7), and 2D‐NMR (Figures S8 and S9) to monitor the formation and dissociation of carbamate in MEA solutions. The results showed significant carbamate (RNH‒CO_2_^−^) formation upon addition of CO_2_ to MEA solutions at the higher end of the investigated pH range. By contrast, samples adjusted to a lower pH (e.g. pH 6.7) showed no detectable carbamate (Table S2). This pH‐dependent stability of carbamate is consistent with many earlier reports [14, 30, 31, 32] and is explained by facile carbamate dissociation via protonation at neutral and low pH.

We utilized this relationship between pH and carbamate content to prepare CO_2_‐loaded samples with respectively high and low carbamate content (see Supporting Information section 5.4). We used these samples in pH‐stat measurements at pH 9, and found that 1 µM PcAmd significantly accelerated CO_2_ release from carbamate‐rich samples (Figure 2A). Conversely, the enzyme did not affect the release rate in carbamate‐lean samples, where CO_2_ was primarily captured as bicarbonate. For the carbamate‐rich samples, the initial CO_2_ release rate was 20% higher in the sample with PcAmd compared to the enzyme‐free sample, and the endpoint level of released CO_2_ after 10 min was about 25% higher. Results from longer experiments (Figure 2B) showed that 1 µM PcAmd reduced the time required to obtain 80% CO_2_ desorption to about half of the enzyme‐free system. The dose‐response curve in Figure 2C showed that the rate increment increased hyperbolically with half‐saturation at about 0.1 µM enzyme.

**FIGURE 2:**
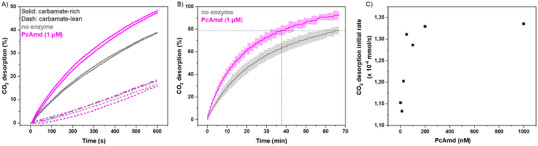
(A) Comparison of pH‐stat data from carbamate‐rich samples (solid lines) and carbamate‐lean samples (dashed lines), (B) Investigation of CO_2_ desorption at a longer timescale, and (C) Effect of enzyme dosage on the CO_2_ release rate. All measurements were conducted and duplicated using 2 mL of 100 mM MEA. The calculation of CO_2_ desorption percentage was described in the Supporting Information (section 5.3).

To investigate the molecular origins of the accelerated CO_2_ release (Figures 2 and S6), we recorded in situ Fourier transform infrared (FTIR) spectra in real time under desorption conditions (see Supporting Information Section 5.5). The mid‐IR region (3000‒800 cm^−1^) was recorded (Figures S11, Panels C/D), and the region 1800‒800 cm^−1^ showed the most relevant bands for an in‐depth analysis. We assigned bands for MEA, [33] carbamate [34], and (bi)carbonate [33, 34, 35] based on the literature and our own controlled analyses to monitor chemical species forming and disappearing during the CO_2_ capture and desorption processes. The absorption bands at 1563 cm^−1^, 1492 cm^−1^, and 1324 cm^−^^1^ indicated the formation of MEA carbamate (c.f. Figures 3A, 3B, and S11). After introducing N_2_ purge gas (0.08 L/min) at *t* = 0 min, the reaction mixture with PcAmd showed increases of new peaks that were assigned as carbonate (1745 cm^−1^, 1462 cm^−1^, and 1380 cm^−1^ v_as_(C‒O) for CO_3_^2−^) and bicarbonate (shoulder peak at 1364 cm^−1^) within 45 min (Figures 3A, and S12) [34, 35]. In addition, a band at 1160 cm^−1^, which we assigned to free MEA based on earlier work, [33] grew commensurate with the (bi)carbonate bands. By contrast, these changes were negligible in the enzyme‐free experiments (Figure 3B), supporting the conclusion that PcAmd can catalyze hydrolysis of carbamate as shown in Scheme 1 (step a).

**FIGURE 3:**
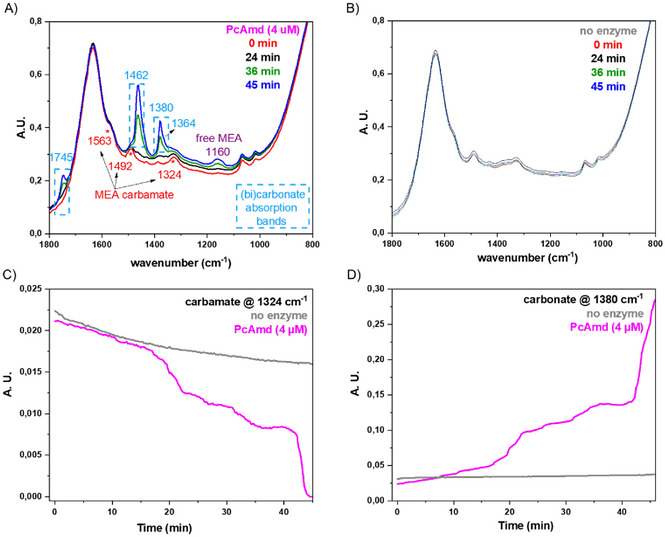
In situ IR spectra of CO_2_ desorption experiments of 25 mL 500 mM MEA with 4 µM PcAmd (Panel A) or without enzyme (Panel B) during purging with N_2_ (0.08 L/min). Trends of height with solvent subtraction at absorption bands 1324 cm^−1^ (Panel C, carbamate) and 1380 cm^−1^ (Panel D, carbonate) were plotted and compared.

To better understand the mechanism of (bi)carbonate accumulation and free MEA regeneration during catalytic desorption, we further analyzed the time‐dependent IR spectra. Specifically, we compared carbamate and carbonate absorption bands after solvent subtraction (Figures 3C, D). We found that the carbamate band at 1324 cm^−1^ reached undetectable levels over a 45 min desorption experiment with 4 µM PcAmd (Figure 3C, pink). For enzyme‐free samples, the carbamate band height remained at about 70% of the initial value (Figures 3C grey, and S13, grey and blue). This enzymatic elimination of the carbamate band was associated with a growth of the (bi)carbonate band at 1364 and 1380 cm^−1^, especially in the amidase‐catalyzed experiment (Figure 3D, pink curve). These observations again supported the view that PcAmd catalyzed the hydrolysis of MEA‐carbamate, and we proposed the mechanism in Scheme 1.

While the IR analysis provided qualitative information, ^1^H NMR and pH‐stat (see Figure S10) suggested a carbamate turnover frequency (TOF) of approximately 1 s^−1^. This corresponds to a specific activity of 1 U/mg, which is within the range of earlier values for amidases acting on urethane and phenylcarbamates [22, 23, 36, 37]. To substantiate these spectroscopic activity data obtained in complex solutions, we also determined the Michaelis‐Menten kinetics of PcAmd using the established Berthelot assay [38] and urethane as substrate. Results in Figure S4 showed *k*_cat_ = 0.25 s^−1^ and *K*_M_ = 0.38 mM. Thus, the TOF in these separate experiments was in the same order of magnitude as the indirect estimates based on NMR data.

It is interesting to note that the effect of PcAmd on the carbamate content (Figure 3C) was more pronounced than the effect on CO_2_ release rates measured in the pH‐stat setup on the same timescale (Figure 2B). The faster breakdown of carbamate according to step *(a)* in Scheme 1 led to an accumulation of (bi)carbonate as shown in Figure 3D. This suggested that when step *(a)* was accelerated by PcAmd, step *(b)* became more critical for the overall rate limitation of the net desorption process. To test this interpretation, we conducted a series of pH‐stat experiments using both PcAmd and carbonic anhydrase (CA) from *P. marina* [39] CA can catalyze step *(b)* in Scheme 1 in both directions [40], and we were hence able to test CO_2_ release under conditions where either step (*a*), step (*b*), or both were enzymatically accelerated. Results in Figure 4A showed enhanced desorption in all three cases. Interestingly, the separate enhancements of the two enzymes were additive (c.f. black and pink curves in Figure 4A). This additivity is in line with the proposed mechanism in Scheme 1 and Figure 4B. As PcAmd catalyzes step *(a)*, this enzyme alone will accelerate carbamate hydrolysis and accumulate bicarbonate (Figures 3C and D). When both enzymes were used, bicarbonate accumulation was alleviated by CA, and an overall increased desorption rate was observed.

**FIGURE 4:**
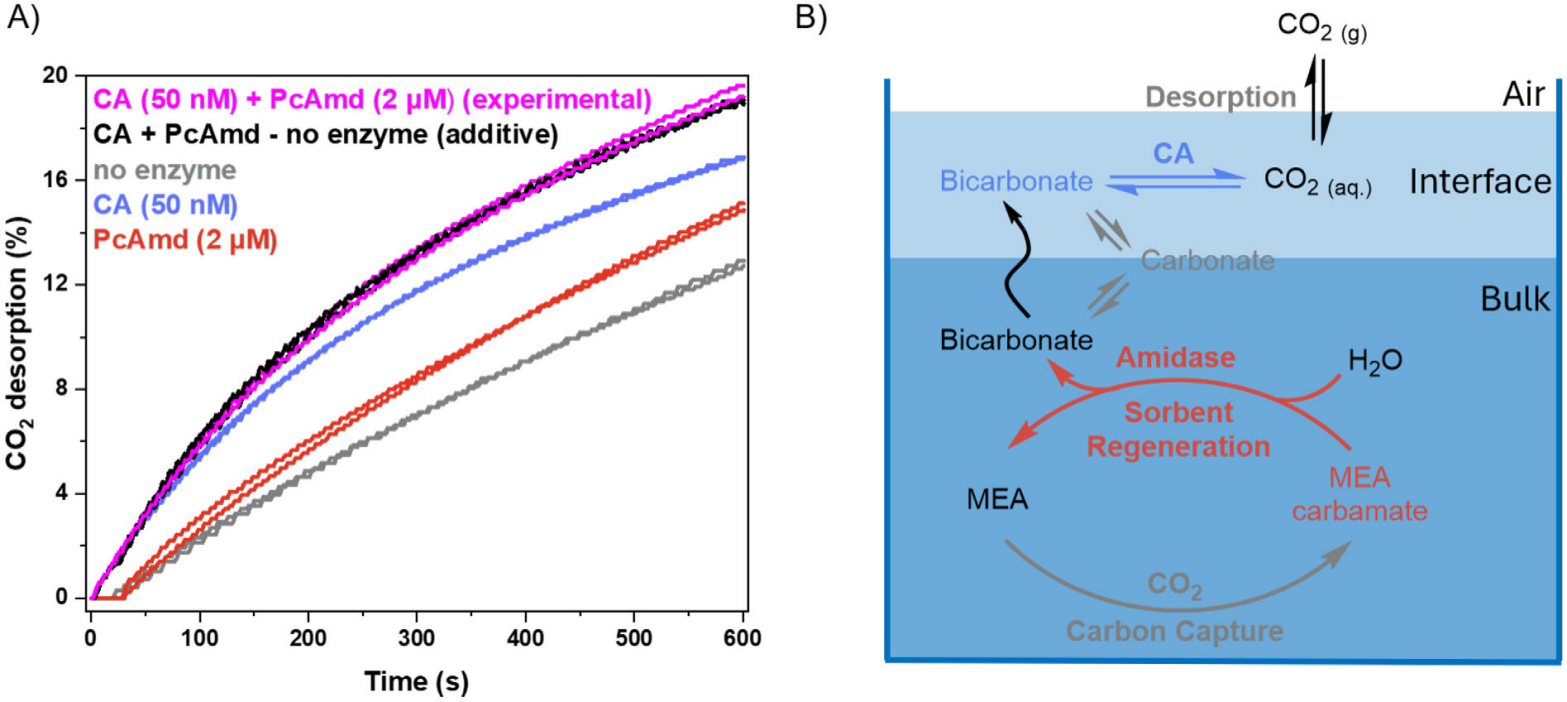
Panel A: CO_2_ release from MEA (500 mM, 2 mL) measured by pH‐stat at pH 9.0. The lines represent duplicate measurements for MEA in enzyme‐free phosphate buffer (grey), buffer supplemented with 50 nM CA (blue), buffer supplemented with 2 µM PcAmd (red) or buffer supplemented with (50 nM CA + 2 µM PcAmd) (pink). The black curve is the sum of the enzyme‐free data and the increments generated by each of the two enzymes. Panel B: proposed mechanism of the additive effect from enzyme cocktails. The effect of the amidase reflects catalysis of step (*A*) in Scheme 1 in the liquid bulk. The effect of CA on the CO_2_ mass transfer results from catalysis of step (*B*) in Scheme 1, especially in a thin film at the gas‐liquid interface [41, 42, 43]

In practice, biocatalytic desorption may be implemented by simply adding the enzyme to the circulating solvent, as is currently done for CA in aqueous carbonate [44, 45]. The high regeneration temperatures (∼120°C) used in present MEA technologies appear incompatible with enzyme stability. However, recent work has suggested that catalyzed desorption may reduce the temperature requirement to 70°C–95°C, [46] which is a realistic range for engineered industrial enzymes. In addition, enzymatic desorption of MEA solvents could be employed with other regeneration principles operating at even lower temperatures. Examples include electrochemical pH swings [47] and vacuum assisted stripping [48]. Another concern regarding practical application is whether activity levels around 1 U/mg as found here are sufficient. We note that even this moderate activity reduced the time required for 80% CO_2_‐release to about half of the time for uncatalyzed desorption (Figure 2B). This appeared as strong proof of concept for biocatalyzed regeneration of MEA solvents, and based on earlier experience, enzyme engineering has a strong potential to improve catalytic performance against non‐native substrates [49].

In conclusion, we identified five novel enzymes that accelerated the release of CO_2_ captured in aqueous MEA, thereby proposing a novel strategy for biocatalytic CO_2_‐stripping. The basic mechanism underlying this effect is enzymatic hydrolysis of the carbamate bond (step (*a*) of Scheme 1). However, the overall kinetics of CO_2_ release is more complex due to couplings within the network of linked equilibria in the amine‐CO_2_‐water system [13, 14]. These couplings were illustrated by the additive effect of PcAmd and CA, suggesting that enzyme cocktails may be promising for future solvent regeneration. If amidases or enzyme cocktails can accelerate CO_2_ desorption, this may lower energy requirements for carbon capture in MEA or other amine‐based sorbents [6, 50, 51]. We propose that engineering and discovery of amidases with higher stability and activity against MEA‐carbamate could become valuable for low‐cost amine‐based carbon capture technologies.

## Author Contributions

**Yang Yang**: methodology, investigation, validation, formal analysis, visualization, writing – original draft, writing – review and editing, data curation, resources. **Stefanie Neun**: resources. **Lea Helena Strother**: resources, writing – review and editing. **Onur Kırtel**: investigation, supervision, visualization, writing – original draft, writing – review and editing, validation, data curation, resources. **Silke Flindt Badino**: supervision, writing – review and editing, formal analysis, methodology, data curation. **Peter Westh**: conceptualization, methodology, visualization, funding acquisition, writing – original draft, writing – review and editing, project administration, formal analysis, supervision, data curation, validation, resources. **Ditte Hededam Welner**: supervision, writing – review and editing, project administration, funding acquisition, writing – original draft. **Jens Preben Morth**: supervision, writing – review and editing. **Ji‐Woong Lee**: methodology, funding acquisition, writing – original draft, writing – review and editing, supervision, project administration. **Jerik Mathew Valera Lauridsen**: validation, data curation, writing – review and editing. **Laura Rotilio**: resources.

## Conflicts of Interest

One of the authors, SN, work for Novonesis, which is a major producer of industrial enzymes.

## Supporting information




**Supporting File**: The authors have cited additional references within the Supporting Information [52, 53, 54, 55, 56, 57, 58].

## Data Availability

The data that supports the findings of this study are available in the supplementary material of this article
