# Supplementary material for: Amidase‐Catalyzed Desorption of CO2 Captured in Aqueous Monoethanolamine (MEA) Solutions
Source: Angew Chem Int Ed Engl. 2026 Jun 26;65(33):e5406475. doi: 10.1002/anie.5406475 (PMC13452561; doi:10.1002/anie.5406475)
Supplement: Supplementary file 1 [file ANIE-65-e5406475-s001.pdf]

Supporting Information for

Amidase-catalyzed Desorption of CO_2 Captured in Aqueous Monoethanolamine (MEA) Solutions

Yang Yang,#[a][b] Onur Kırtel,#[c] Silke Flindt Badino,[a][b] Lea Helena Strother,[c] Laura Rotilio,[a] Jerik Mathew Valera Lauridsen,[b][e] Stefanie Neun,[d] Jens Preben Morth,[a] Ji-Woong Lee,[b][e] Ditte Hededam Welner,[c] and Peter Westh*[a][b]

# Equal contribution

[a] Protein and Enzyme Technology, Department of Biotechnology and Biomedicine, Technical University of Denmark, 2800 Kgs Lyngby, Denmark E-mail: petwe@dtu.dk

[b] The Novo Nordisk Foundation CO_2 Research Center (CORC), Aarhus University, Gustav Wieds Vej 10, Aarhus 8000, Denmark

[c] The Novo Nordisk Foundation Biotechnology Research Institute for the Green Transition, Technical University of Denmark, Søltofts Plads 220, 2800 Kgs. Lyngby, Denmark

[d] Novonesis, Biologiens Vej 2, 2800 Kgs Lyngby, Denmark

[e] Department of Chemistry, University of Copenhagen, Universitetsparken 5, 2100 Copenhagen Ø, Denmark

Table of Contents

1. Sequence mining for amidases S3

2. *In silico* structural analyses S6

3. Boltz-2 structure predictions and DiffDock-L docking analyses S8

4. Enzyme production and purification S8

5. Methods and supplementary results for amidase characterization S10

5.1 Thermostability of amidases using NanoDSF S10

5.2 pNPB assay and Berthelot assay S12

5.3 pH-stat analysis S12

5.4 pH-dependent NMR analysis of MEA-CO_2 mixture in D_2O S13

5.4.1 pH-stat/^{1}H NMR analysis S16

5.5. Enzymatic CO_2 desorption experiments with *in situ* FTIR spectroscopy S17

6. Rough calculations of protons consumed for one molecule of CO_2 desorption from MEA solutions at pH 9.0 ....S20

References S22

1. Sequence mining for amidases

We have carried out sequence mining using four characterized amidase/urethanase sequences as templates: An ethanol-resistant, urethane (ethyl carbamate)-active amidase from *Agrobacterium tumefaciens* d3 (AAK28498.1)[24] and three polyurethane-degrading urethanases from uncultured clones (WBR49956.1, WBR49957.1, WBR49958.1).[23] In order to find proteins that are potentially organic solvent resistant, alkalistable or thermostable, the protein-protein BLAST search was restricted to sequences from the *Bacillaceae* family due to the widespread presence of extremophilic microorganisms in that taxon.[52] The selection criteria were i) the presence of the catalytic triad Lys98-*cis*Ser193-Ser197 (residues numbered according to *A. tumefaciens* d3 amidase) that is strictly conserved within the AS superfamily enzymes, ii) minimum 40% sequence identity with any of the query sequences, iii) one sequence from each species and iv) alkaliphilic (high pH resistance), halophilic (tolerance to organic solvents) or thermophilic (thermostability) origin of the host organisms. Sequences meeting these criteria were used to build a phylogenetic tree on iTOL.[53] At least two distinct sequences from each clade on the tree were chosen for cloning and enzyme production.

An overview of the sequences mined in this study can be found in Table S1:

Table S1 *Bacillaceae amidase sequences mined in this study*

	**Genbank accession**	**Organism**	**Query cover**	**% identity**
**Query**	AAK28498.1	*Agrobacterium tumefaciens* d3		
**Hits**	ODA15694.1	*Geobacillus thermoleovorans*	97%	49.8%
	WP_232722101.1	*Geobacillus* sp. Manikaran-105	97%	49.8%
	GAD14385.1	*Geobacillus kaustophilus* GBlys	97%	49.6%
	WP_090947909.1	*Parageobacillus thermantarcticus*	97%	49.4%
	WP_125010964.1	*Parageobacillus thermoglucosidasius*	98%	48.02%
	WP_248893798.1	*Bacillus methanolicus*	97%	49.20%
	WP_017728720.1	*Halalkalibacterium ligniniphilum*	96%	47.70%
	WP_144449828.1	*Halalkalibacter nanhaiisediminis*	98%	47.94%

	WP_289475385.1	*Fictibacillus enclensis*	97%	49%
	WP_183251019.1	*Anoxybacillus tepidamans*	96%	49.3%
	WP_085031211.1	*Halobacillus mangrovi*	97%	49.4%
	WP_061579417.1	*Parageobacillus caldoxylosilyticus*	97%	48.01%
	WP_078427407.1	*Alkalihalobacterium alkalinitrilicum*	96%	47.5%
	WP_076767038.1	*Domibacillus epiphyticus*	96%	47.41%
	WP_155221332.1	*Terrilactibacillus tamarindi*	96%	46.8%
	WP_307320820.1	*Evansella vedderi*	96%	45.22%
	SDE54970.1	*Priestia aryabhattai* B8W22	96%	44.62%
**Query**	WBR49956	Uncultured clone 1 (UMG SP1)		
**Hits**	WP_320379496.1	*Virgibacillus soli*	52%	41.13%
	WP_215065119.1	*Oceanobacillus* sp. ISL-73	52%	40%
	WP_017797476.1	*Oceanobacillus kimchii*	52%	40%
	WP_257351917.1	*Pseudalkalibacillus decolorationis*	52%	40.09%
	WP_243602259.1	*Virgibacillus halodenitrificans*	44%	43.59%
	WP_102027613.1	*Salirhabdus* sp. Marseille-P4669	40%	42.94%
	WP_115823524.1	*Halobacillus trueperi*	37%	40.85%
**Query**	WBR49957	Uncultured clone 2 (UMG SP2)		
**Hits**	WP_091275266.1	*Alteribacillus persepolensis*	57%	40%
	WP_227938354.1	*Alkalihalobacillus deserti*	51%	40.17%
	WP_197226046.1	*Bacillus xiamenensis*	38%	41.62%
	WP_144088922.1	*Allobacillus salarius*	39%	40.23%
	WP_117157030.1	*Paraliobacillus quinghaiensis*	42%	41.49%
	WP_102261311.1	*Mesobacillus jeotgali*	38%	40.70%
	WP_114374250.1	*Salicibibacter kimchii*	42%	40.31%
	WP_244682417.1	*Lederbergia wuyishanensis*	38%	41.28%
	WP_245690120.1	*Sediminibacillus albus*	41%	40.54%
	WP_271340263.1	*Bacillus changyiensis*	39%	41.01%

	WP_182201408.1	*Paraliobacillus salinarum*	42%	40.31%
	WP_143895827.1	*Radiobacillus deserti*	43%	40.62%
**Query**	WBR49958	Uncultured clone 3 (UMG SP3)		
**Hits**	WP_200088868.1	*Salicibibacter cibi*	52%	41.74%
	WP_240377012.1	*Bacillus piscicola*	71%	42.54%
	WP_217067565.1	*Evansella tamaricis*	42%	40.64%
	WP_089739532.1	*Gracilibacillus ureilyticus*	42%	42.55%
	WP_119546493.1	*Bacillus salacetis*	41%	40.76%
	WP_091483673.1	*Gracilibacillus orientalis*	42%	40.31%
	WP_163583185.1	*Gracilibacillus saliphilus*	42%	40.96%
	WP_153835557.1	*Gracilibacillus thailandensis*	42%	40.43%
	WP_018932225.1	*Gracilibacillus lacisalsi*	42%	40.43%
	WP_109985656.1	*Gracilibacillus dipsosauri*	42%	40.10%
	WP_017185211.1	*Alkalibacillus haloalkaliphilus*	43%	40.53%
	WP_237582668.1	*Pontibacillus yanchengensis*	41%	42.70%
	WP_117168238.1	*Paraliobacillus sediminis*	42%	40.64%
	WP_027963642.1	*Halalkalibacillus halophilus*	39%	41.95%
	WP_097158554.1	*Bacillus oleivorans*	38%	40.35%
	WP_066173926.1	*Bacillus marinisedimentorum*	38%	40.35%
	WP_204438941.1	*Amphibacillus cookii*	43%	40%
	WP_066264355.1	*Heyndrickxia acidicola*	38%	42.77%
	WP_113971106.1	*Rosellomorea aquimaris*	38%	40.12%
	WP_229675574.1	*Halolactibacillus alkaliphilus*	40%	40%
	WP_062442892.1	*Thalassobacillus devorans*	43%	40.51%
	WP_121440117.1	*Salisediminibacterium halotolerans*	43%	40.62%
	WP_188632940.1	*Lentibacillus kapialis*	67%	40.53%
	WP_077735767.1	*Bacillus sonorensis*	42%	42.55%
	WP_320156914.1	*Metabacillus indicus*	38%	41.38%
	WP_251430863.1	*Mesobacillus maritimus*	35%	41.03%
	WP_046179569.1	*Domibacillus tundrae*	30%	40%

	WP_148958683.1	*Bacillus swezeyi*	38%	44.19%
	WP_281351762.1	*Alkalicoccus luteus*	40%	41.21%

2. *In silico* structural analyses

We have conducted *in silico* structural analyses to elucidate the overall structural properties of the amidases.

We have used the NeuroSnap web server and PyMOL 3.0.3 (Schrodinger, LLC) for all *in silico* investigations.[54] Amidase structures with MEA carbamate (2-hydroxyethylcarbamic acid) as the ligand were predicted via Boltz-2.[55] Configuration parameters were as followed: Diffusion samples: 5, affinity diffusion samples: 10, multiple sequence alignment mode: mmseqs2_uniref_env, molecular weight correction: false, recycle numbers: 6, pocket restraints: none, residue modifications: none, sampling steps: 200, affinity sampling steps: 300, step scale: 1.638, use inference time potentials: false. For DiffDock-L docking analyses we have used the PcAmd structure predicted by Boltz-2 without MEA carbamate.[56] Parameters were same as above except for affinity diffusion samples (5) and affinity sampling steps (200). The resulting protein structure was used to dock MEA carbamate with 150 sample numbers.

As seen in Figure S1a, amidases from clades A and D both share a canonical helix-turn-helix motif on their N-termini, though their overall folded states seem vastly different with a root mean square deviation (RMSD) of 3.472 Å between AdAmd and PcAmd. The closest homologue of PcAmd with an experimental 3D structure is an amidase from *Rhodococcus* sp. N-771 (PDB ID: 3A1K, 49.3% sequence identity, RMSD 1.492 Å), which forms a homodimer through a domain swap of its N-terminus (Figure S1b). Boltz-2 prediction for a PcAmd dimer yields a homodimeric structure with a fold that is highly similar to 3A1K’s (Figures S1c-d, RMSD between the dimers: 1.763 Å). It was also observed during our experiments that PcAmd fractions completely remained in the retentate of 100 kDa buffer exchange centrifuge tubes, overall indicating a possible tertiary structure of a homodimer.

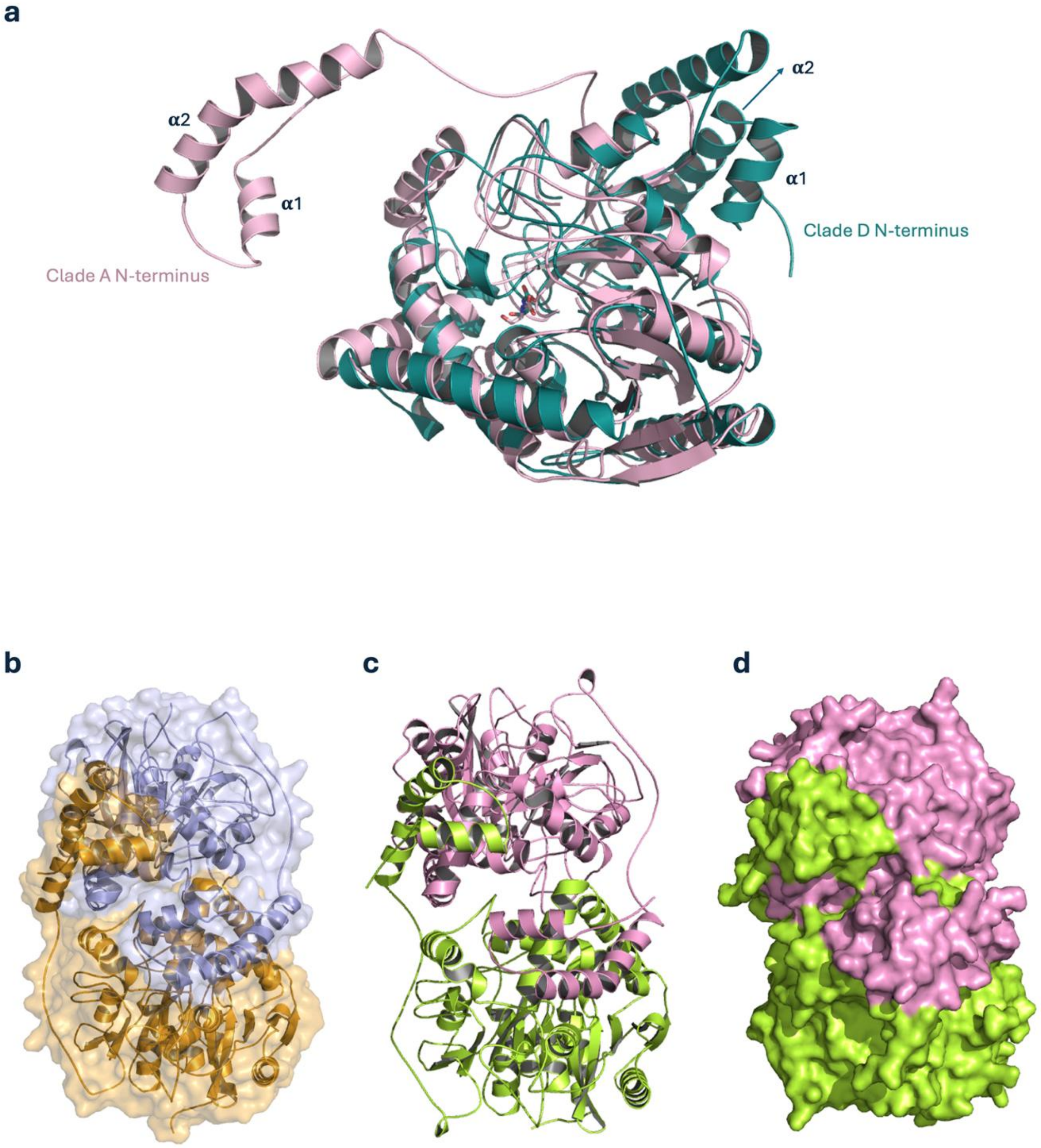

Figure S1. *Structural analyses of clade A and D amidases. a) Alignment of Boltz-2 structures of PcAmd (pink, complex pLDDT: 0.966) and AdAmd (teal, complex pLDDT: 0.970) along with MEA carbamate at the active sites (ligand iPTM: 0.916 and 0.890, respectively), b) Homodimer of Rhodococcus sp. N-771 amidase (PDB ID: 3A1K). c) Cartoon and d) Surface representations of PcAmd homodimer as predicted via Boltz-2 (complex pLDDT: 0.966).*

3. Boltz-2 structure predictions and DiffDock-L docking analyses

Boltz-2 structure predictions and DiffDock-L docking analyses with MEA carbamate (2-hydroxyethylcarbamic acid) as the ligand suggest that MEA carbamate is hydrolyzed to release CO_2 via the classical AS superfamily mechanism: S195 (–OH) in PcAmd is predicted to be situated 2.4 – 2.6 Å away from the carbonyl carbon of MEA carbamate, at which it performs a nucleophilic attack. This is catalyzed by the deprotonation of K96 (–NH_2), the general acid/base, while S171 (–OH) acts as a proton shuttle between the two residues (Figure S2), resulting in CO_2 and ethanolamine release.

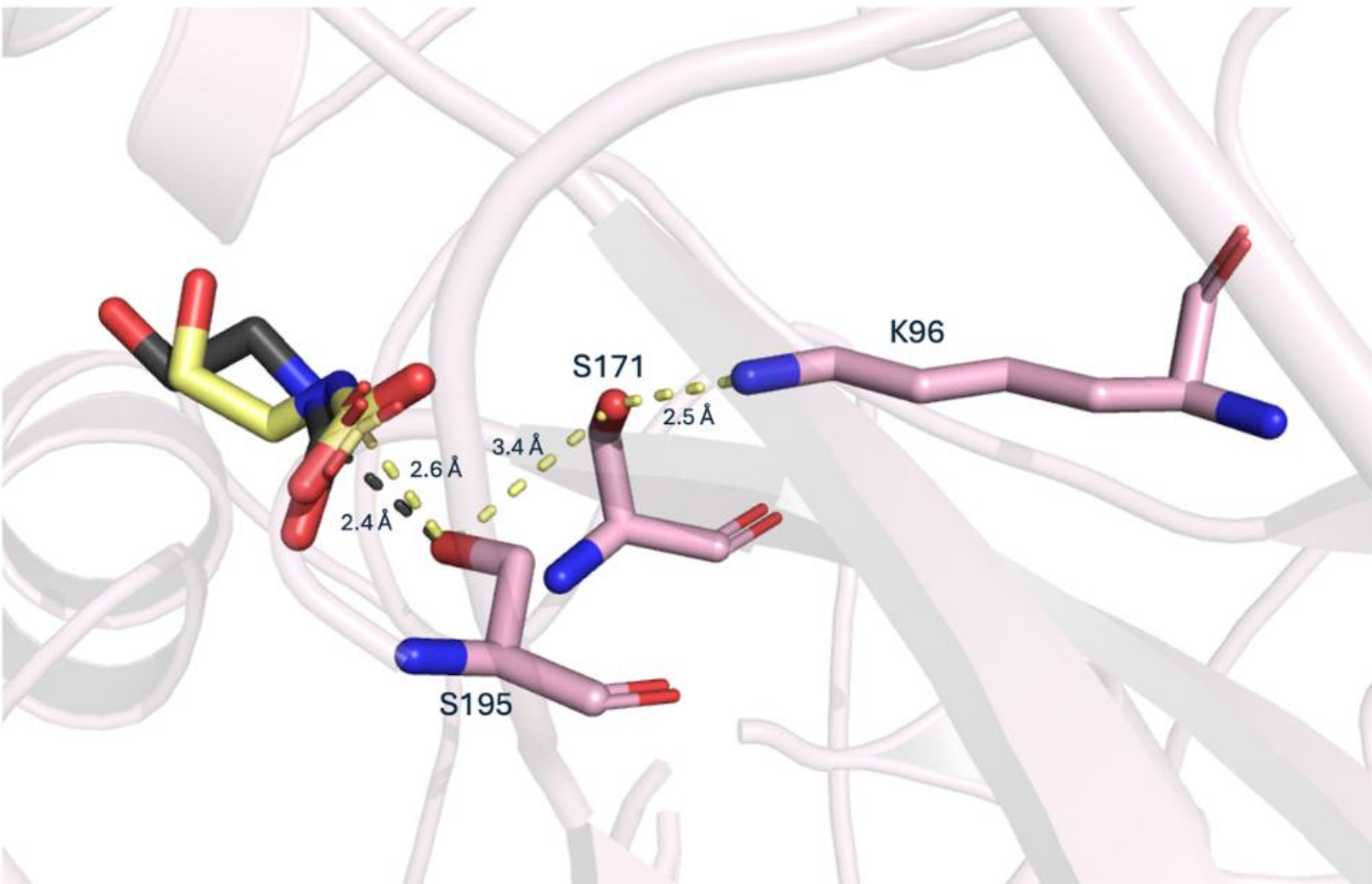

Figure S2. *Boltz-2 structure prediction for the active site of PcAmd along with MEA carbamate. The distance between the nucleophile S195's –OH group and the carbonyl carbon of MEA carbamate were predicted to be 2.4 Å (DiffDock-L, black sticks) or 2.6 Å (Boltz-2, yellow sticks). DiffDock-L quality scores were as follows: 0.55 Confidence, -2.51954 SMINA Minimized Affinity and 0.659 SMINA Minimized RMSD.*

4. Enzyme production and purification

Most chemicals and reagents used for enzyme production and purification were from Sigma-Aldrich or Merck at the highest purity available. Chosen native gene sequences were cloned by Biomatik USA, LLC into pET-28a(+) expression vectors between the *NcoI* – *XhoI* restriction sites with N-terminal 6xHis-tags and TEV cleavage sites with linkers

(MGSSHHHHHHSSGENLYFQGSS-) and purchased. A standard heat shock transformation method was applied to One Shot™ BL21 Star™ (DE3) Chemically Competent *Escherichia coli* (Thermo Fisher Scientific) cells to obtain transformants, which were incubated overnight at 37 °C on LB plates with 50 µg/mL kanamycin as selection marker. Positive clones were picked the next day and grown in 5 mL of 2xYT media with 50 µg/mL kanamycin (2xYT-Kan) in 50 mL Falcon tubes at 37 °C and 200 rpm until OD_{600} reached ca. 0.7, which were then stored in 20% (v/v) glycerol at –70 °C for further experiments.

Enzyme production was initiated with precultures growing overnight in 7.5 mL of 2xYT-Kan media in 50 mL Falcon tubes at 37 °C and 200 rpm. 500 mL of 2xYT-Kan media supplemented with 0.005% Antifoam 204 (Sigma-Aldrich) in 2 L baffled Erlenmeyer flasks were inoculated with 1.5% (v/v) preculture and left to grow under the same conditions until OD_{600} exceeded 0.8. Protein expression was initiated with the addition of isopropyl β-D-1-thiogalactopyranoside (IPTG) to a final concentration of 250 µM, followed by reducing the temperature to 18 °C for overnight incubation. Cells were pelleted the next day via centrifugation at 4,700 rpm for 30 min at 4 °C. Cell pellets were resuspended in a lysis buffer containing 50 mM disodium phosphate, 300 mM NaCl, 20 mM imidazole, 1 mg/mL lysozyme (from chicken egg white, Merck) and 1 cOmplete™, EDTA-free Protease Inhibitor Cocktail tablet (Roche) per 50 mL (pH 7.6). Cell suspensions on ice were disrupted via ultrasonication on a VCX-130 Ultrasonic Processor (Medline Scientific) with a 30 s on/30 s off cycle at 60% nominal power for 12 min in total. The lysates were centrifuged at 14,500 *x g* for 55 min at 4 °C and the resulting supernatants were filtered through 0.45 µm syringe filters before purification.

A HisTrap FF 1 mL (Cytiva, USA) column on an ÄKTA Pure™ system was used for protein purification with two buffers at pH 7.6: Buffer A (50 mM disodium phosphate, 300 mM NaCl, 20 mM imidazole) and Buffer B (50 mM disodium phosphate, 300 mM NaCl, 500 mM imidazole). The column was equilibrated with 10 column volumes (CV) of Buffer A. After sample application, the column was washed with 20 CV of 94.8% Buffer A + 5.2% Buffer B. Proteins were eluted from the column with the following program: 0% to 40% linear increase to Buffer B in 20 CV, 100% Buffer B for 5 CV, followed by 100% Buffer A for 5 CV. The flow rate was 1 mL/min throughout the process. Fractions of 1 mL were collected in 96-deepwell plates. The amount and purity of the target proteins were determined with absorbance readings on a NanoDrop™ 2000/2000c spectrophotometer (Thermo Fisher Scientific) and sodium dodecyl sulfate – polyacrylamide gel electrophoresis (SDS-PAGE; NuPAGE™ Bis-Tris Mini

Protein Gels, 4–12%, 1.0–1.5 mm, Thermo Fisher Scientific), respectively. Fractions of interest were pooled, and buffer exchanged three times against a storage PBS buffer containing 25 mM disodium phosphate and 150 mM NaCl (pH 7.6) using Amicon Ultra 15 mL 50 kDa MWCO centrifugal filters (Sigma-Aldrich) to remove imidazole. Purified protein fractions were aliquoted, and flash frozen in liquid nitrogen to be stored at –70 °C for further experiments.

5. Methods and supplementary results for amidase characterization

5.1 Thermostability of amidases using NanoDSF

The thermostability of the amidases were measured on a Prometheus Panta instrument from NanoTemper. The amidases were scanned from 25 to 85 ºC with a scan rate of 1 ºC/min.

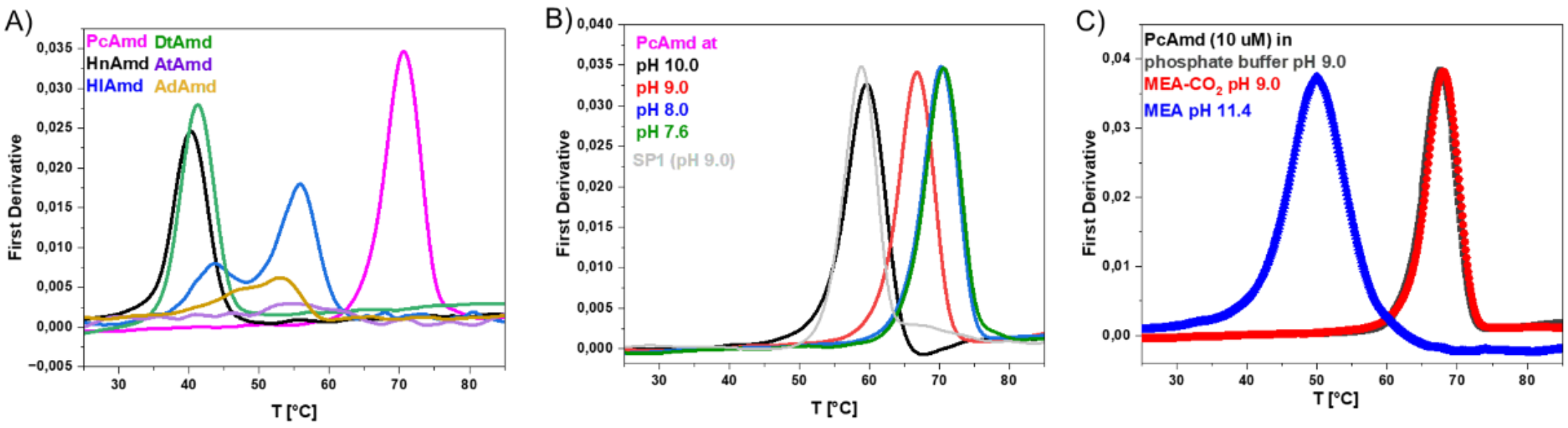

Figure S3. *NanoDSF measurements were conducted with amidases (10 µM in phosphate buffer) at pH 7.6 (A), PcAmd at different pHs and comparison with SP1 at pH 9.0 (B), and the effect of MEA (100 mM) with CO_2 at pH 9.0, and without CO_2 at pH 11.4 (C). All measurements were duplicated.*

5.2 Activity and stability tests on the Berthelot assay and pNPB assay

5.2.1 The Berthelot assay and Michaelis Menten kinetics of PcAmd.

Kinetic data of PcAmd on urethane was obtained by the Berthelot colorimetric assay.[38] The enzyme concentration was 100 nM of PcAmd and substrate concentration ranged from 0 - 50 mM urethane. Reactions of 200 µL in 25 mM Na_2HPO_4 (pH 8.0) were run at 50 °C for 15 min (30 min for the lower substrate concentration range). Reactions were stopped via the addition of an equal volume of 10% (w/v) trichloroacetic acid, incubated at room temperature for 5 min and then neutralized via the addition of stoichiometric amount of NaOH before the detection

of free ammonia via the Berthelot assay. Absorbance values collected at 625 nm were converted to ammonia concentration via the standard curve obtained (Figure S4).

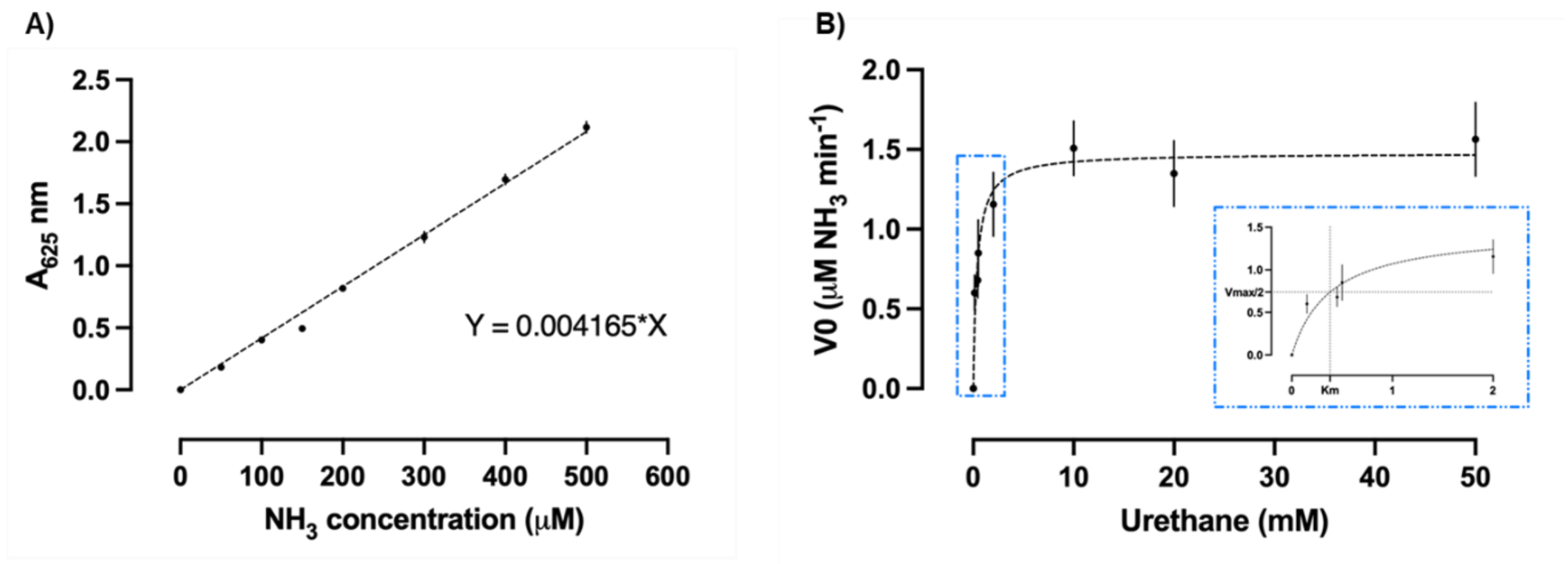

Figure S4. Michaelis-Menten kinetics of PcAmd on urethane. NH_3 release was measured via the Berthelot colorimetric assay and quantified against a NH_4Cl standard curve (Panel A, R^2 = 0.9952). Panel B shows the initial, steady-state rates against the substrate concentration and the dashed line represents a Michaelis-Menten fit. The parameters were K_M = 0.38 mM (95% CI = 0.2372 to 0.5858 mM), k_{cat} = 0.25 s^{-1} (95% CI = 0.225 to 0.268 s^{-1}), R^2 = 0.8895. Data points represent mean ± SD of triplicates and the inset show a magnification of the low-concentration range.

5.2.2 Kinetic stability of PcAmd

The kinetic stability of PcAmd was investigated by testing residual activity following exposure to predefined temperatures. PcAmd (4 µM) in phosphate buffer at pH 9.0 was preincubated at 25 ºC, 40 ºC and 60 ºC for 0, 0.5, 1, 2, 4, 6, 24, and 48 h. The stock solutions were then removed from the incubators and cooled down on ice. The hydrolysis pf pNPB was measured after 30 minutes at room temperature, using 10 mM pNPB and 2 µM enzyme and a total volume of 200 µL. The release of *p*-nitrophenol was measured spectrophotometrically at 405 nm. Absorbance values were converted to the remaining activity (%), and plotted as a function of incubation time (h) in Figure S5.

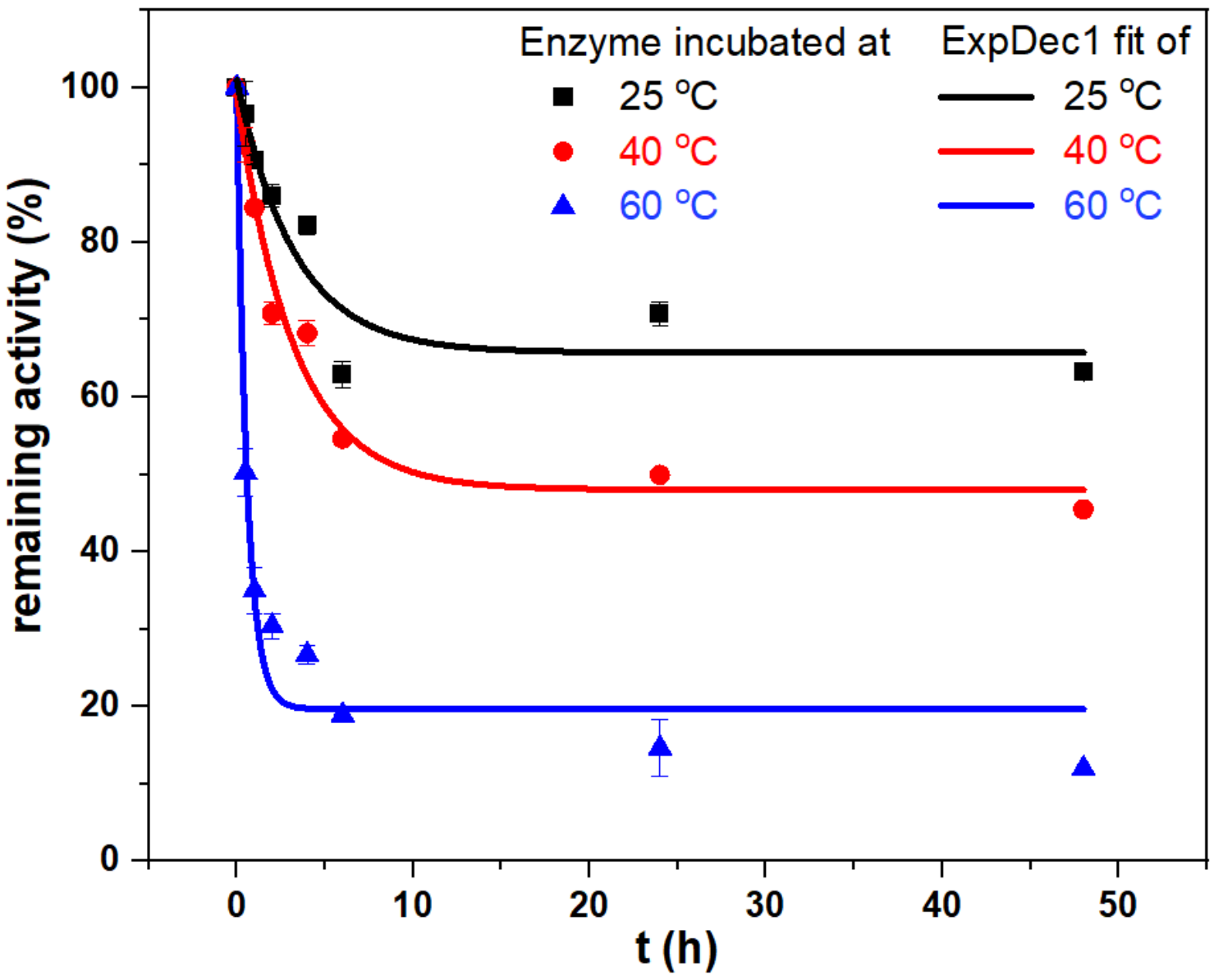

Figure S5. Kinetic stability of PcAmd at different temperatures. The half-time of activity loss for PcAmd ($t_{½}$) was derived from exponential fits, and we found $t_{½}$=2.3 h (25°C), $t_{½}$=2.2 h (40 °C), and $t_{½}$=0.4 h (60 °C). All data points represent averages of triplicate measurements.

5. 3 pH-stat analysis

pH-stat analysis was performed on a SI Analytics Titroline 7000 workstation equipped with a N6000 or Z453 pH electrode. Monoethanolamine (MEA) from Merck was used as sorbent in all experiments. Prior to the desorption analyses the MEA solution was bubbled with CO_2 to reach pH 8.8. Hereafter, the solution containing MEA carbamate was purged with nitrogen to initiate CO_2 desorption. During this phase the pH was kept constant at pH 9.0 using the pH-stat function where HCl was titrated into the solution to keep pH constant. The consumption of HCl solution was converted to CO_2 desorption (%, the amounts of CO_2 released divided by the total amounts of CO_2 captured in MEA solution at pH 9.0) and plotted (see Scheme S1 and S2 in Section 6 for further details). For CO_2 desorption experiments with carbamate-lean samples (Figure 2A), CO_2 gas was bubbled into MEA solutions to pH 6.7, where no carbamate exists according to Figure S5. Afterwards, N_2 purge gas was turned on and pH increased to 8.5. Hereafter, pH-stat analysis was performed at pH 9.0.

Experimental conditions varied between measurements and details about sorbent concentration, enzyme load, reaction volume, and timescale are shown in individual figure legends.

All experiments were performed at room temperature and gas cylinders were purchased from Air Liquide CO_2 (purity ≥ 99%) and N_2 (purity ≥ 99%). Gas flows were regulated by SFC5500 Series Multi-Gas Mass Flow Sensors.

All available amidases were screened using a 10 min desorption screen.

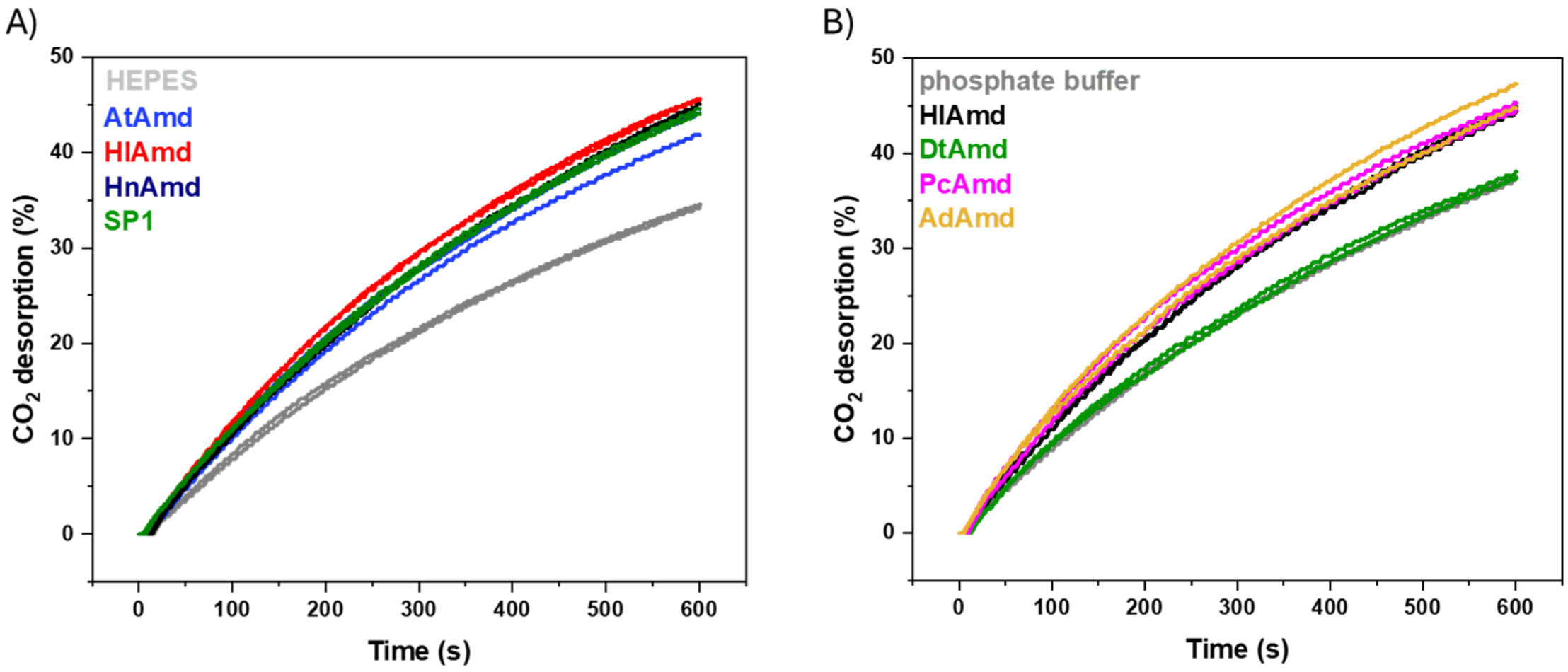

Figure S6. *pH-stat screening of different amidases/urethanases. 100 mM MEA aqueous solution was used, and the enzyme concentration was 1 µM. The reaction volume was 2 mL, and nitrogen was purged at a flow rate of 60 mL/min.*

5. 4 pH-dependent NMR analysis of MEA-CO_2 mixture in D_2O

Liquid ^{1}H and ^{13}C NMR spectra were recorded with a 500 MHz Ultrashield Plus 500 spectrometer and 125 MHz on a Bruker Avance 3 spectrometer with a BBFO probe. A solution of 20 mM MEA in D_2O, was slowly bubbled with CO_2 (purity ≥ 99%, balloon) while monitoring the pH changes at room temperature. Aliquots (0.5 mL) were sampled at different pH-values for analysis. The yields of MEA–CO_2 adduct (or carbamate anion) is shown in Table S2. The values were calculated based on the integration of protons (*O*–**CH₂**–CH₂–NH–COO$^-$, the smaller peak at 3.6 ppm in Figure S5), which was divided by the sum of the integrations of the *O*–**CH₂** protons showing up between 3.5 ppm and 4.0 ppm in each ^{1}H NMR spectrum. The yields of carbamate were calculated considering the ion pair consisting of two molecules of MEA.

Table S2. *^{1}H NMR yields of MEA carbamate at different pHs in D_2O*

pH	^{1}H NMR Yield Carbamate[a]
11.32	0, No CO_2 bubbling
9.98<br>After 18 h[b]	48%<br>27%
8.70<br>After 18 h[b]	54%<br>34%
7.84	34%
7.43	29%
6.76	Not detectable

a, The fraction of carbamate ion pairs was calculated. b, ^{1}H NMRs of the same sample were recorded again after 18 h and around 20% of the carbamate decomposed in the NMR tubes during that time.

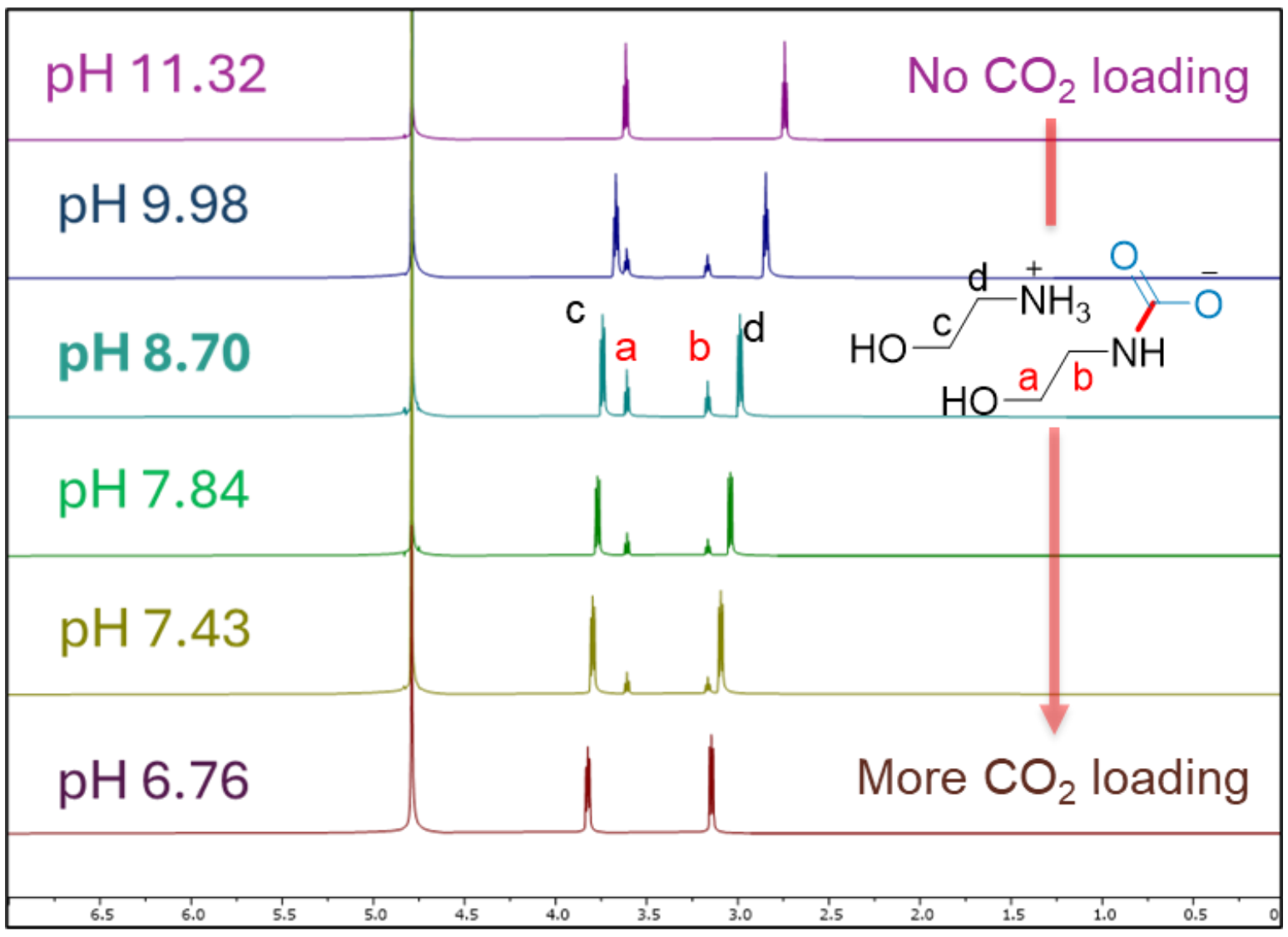

Figure S7. *pH-dependent ^{1}H NMR spectra of MEA (20 mM) and CO_2 mixture in D_2O. Protons c and d of the protonated MEA were overlapped with those of neutral MEA molecules. The carbamate yield peaked around pH 8.7, whereas more CO_2 loading resulted in carbamate decomposition as the media become more acidic. At pH 6.76, no carbamate was detectable.*

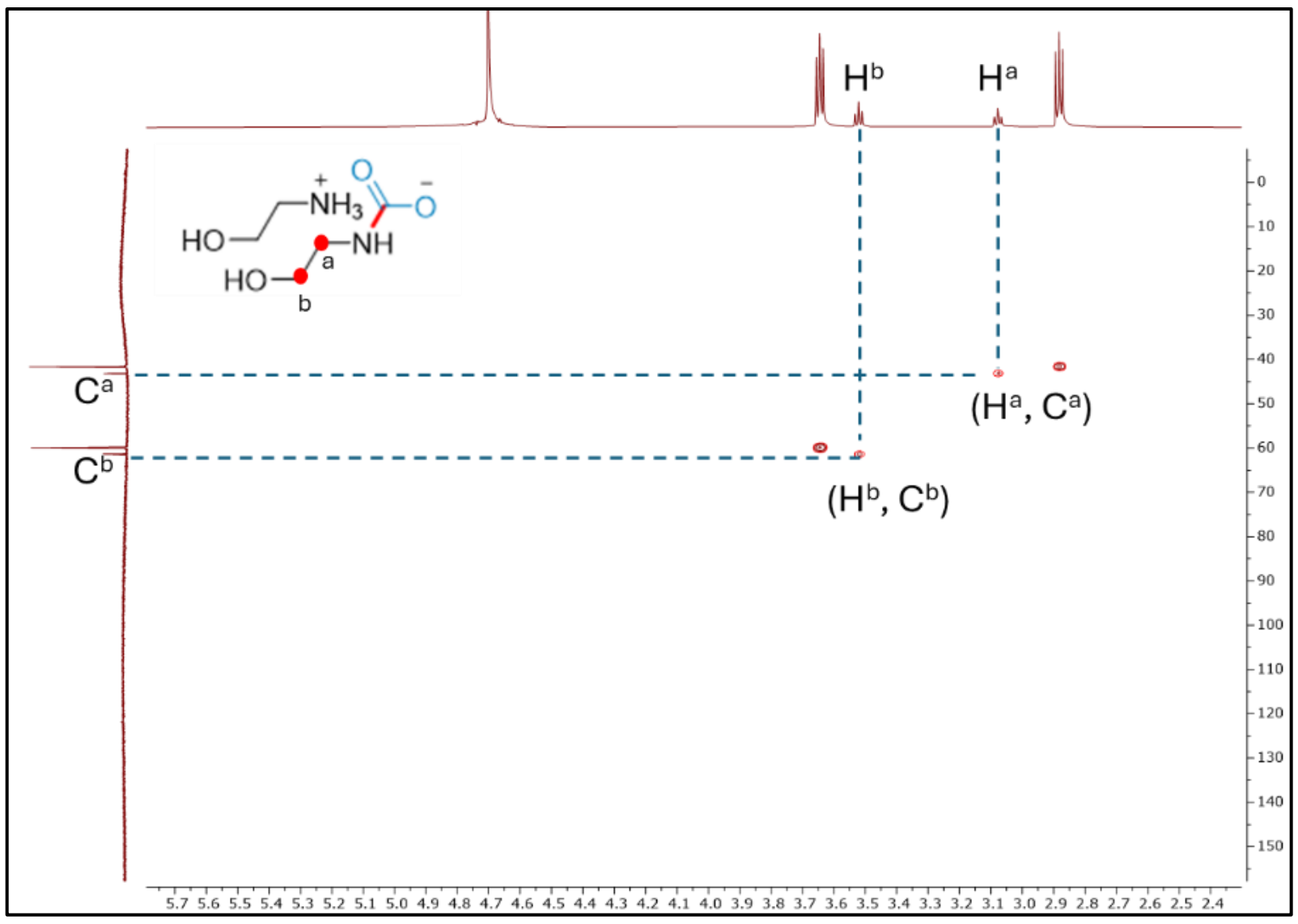

Figure S8. *HSQC (Heteronuclear Single Quantum Coherence) 2D-NMR spectroscopy showing the correlations between carbon and its attached protons of the carbamate anion.*

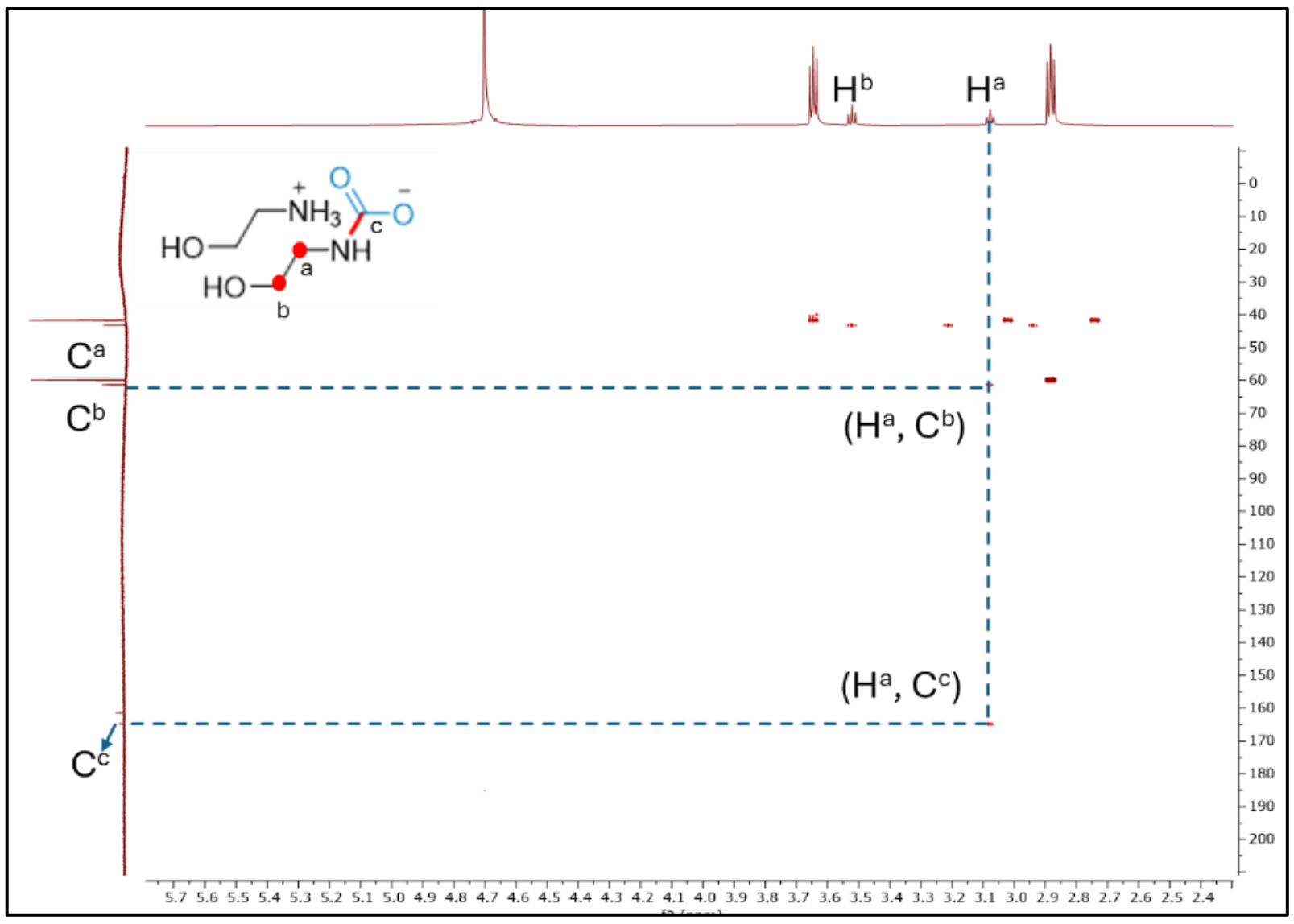

Figure S9. *HMBC (Heteronuclear Multiple Bond Correlation) 2D-NMR spectroscopy showing correlations between carbons and protons that are attached to adjacent carbons of the carbamate anion. The H^a-C^c correlation supported the formation of MEA carbamate.*

5.4.1 pH-stat/^{1}H NMR analysis

We also performed long pH-stat experiments combined with ^{1}H NMR analysis. Here aliquots of 0.1 mL from the pH-stat measurement were taken and diluted 5 times in D_2O. The carbamate content was measured shortly after using ^{1}H NMR.

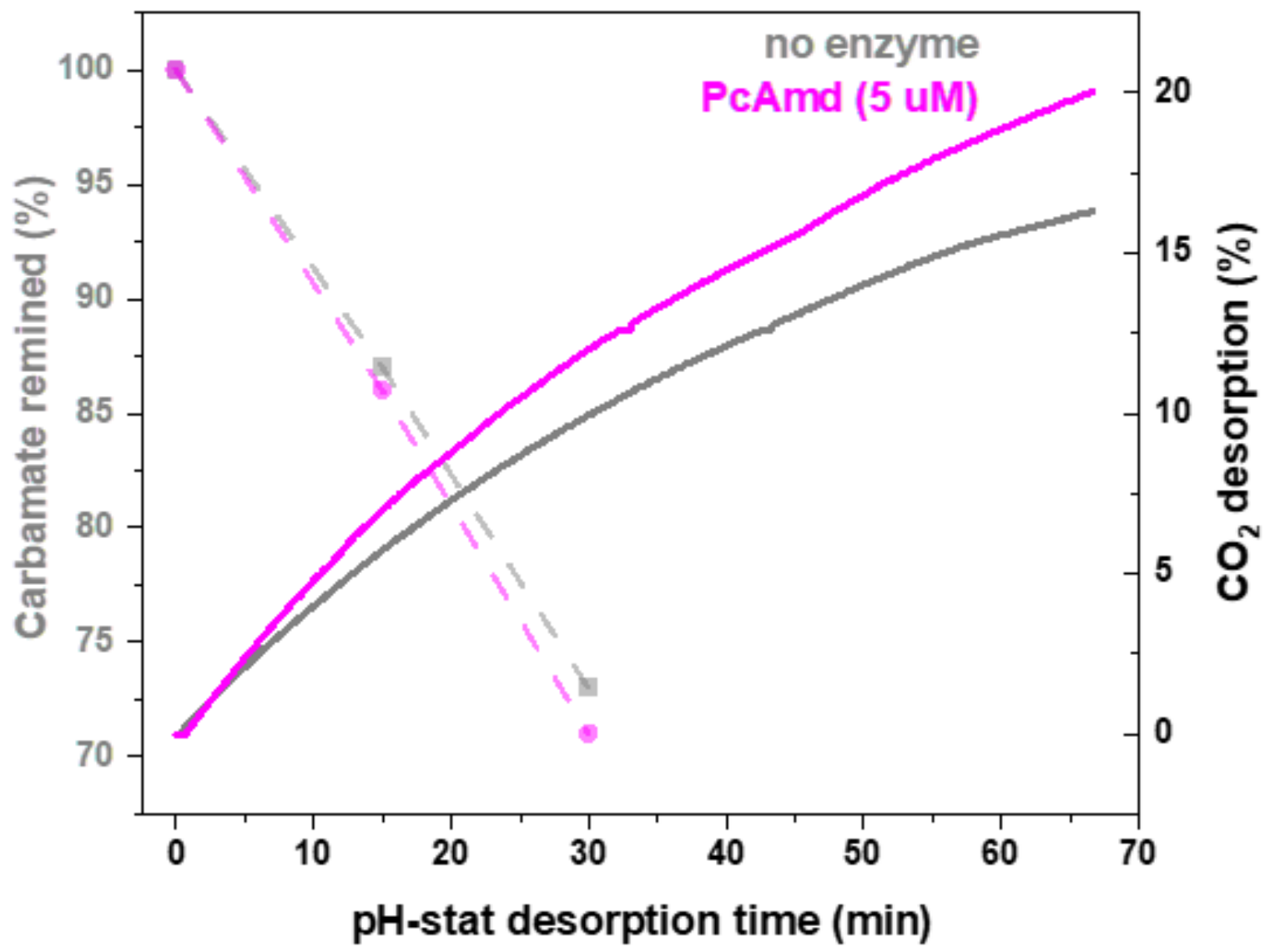

Figure S10. *Solid lines presented pH-stat data of CO_2 desorption from MEA solution (500 mM, 25 mL) with or without PcAmd (5 µM). Dash lines showed carbamate remained (normalized, %) by ^{1}H NMR measurements of aliquots that were sampled at t = 0 min, 15 min, and 30 min, and diluted 5 times with D_2O.*

5. 5. Enzymatic CO_2 desorption experiments with *in situ* FTIR spectroscopy

FTIR spectroscopy was performed in a three-neck round bottom flask equipped with a ReactIR 15 probe and a pH electrode. A 25 mL solution of 500 mM or 1 M MEA was stirred during the collection of spectral data. First, CO_2 was bubbled into the system using a balloon (purity ≥ 99%) while the pH decrease was monitored. At pH 8.7 the gas was shifted to N_2 (0.08 L/min), the desorption experiment started upon addition of enzyme (or enzyme-free phosphate buffer for control experiment). The in-situ Fourier transform infrared (FTIR) spectra of the solution was recorded in real-time over the whole period of CO_2 capture and desorption process. The experiments were performed at 25 °C using a water bath to control the temperature. Figure S9 shows how the carbamate was formed and slowly degraded when purging with nitrogen. The experiments were performed with or without 4 µM PcAmd.

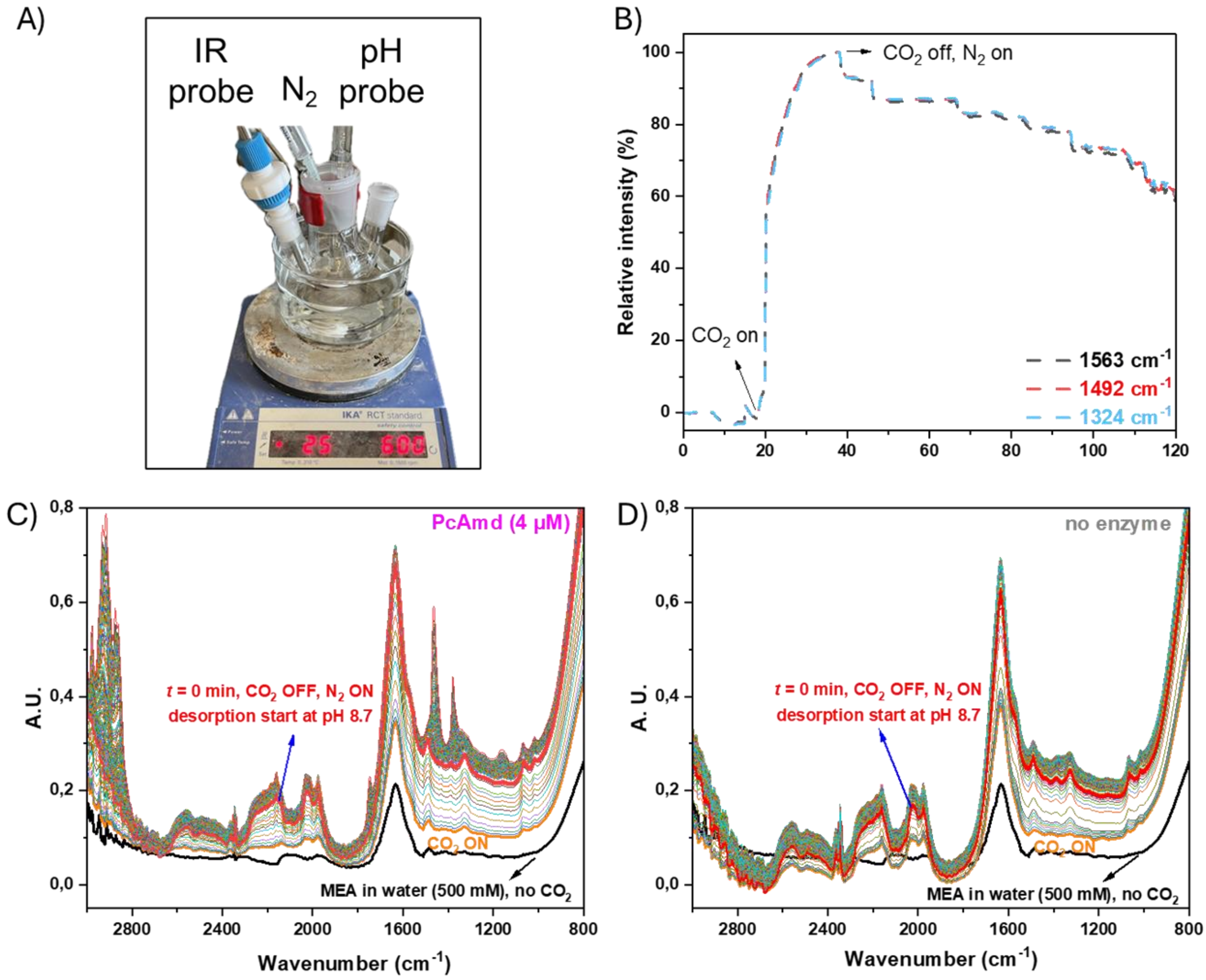

Figure S11. *In situ FTIR experiments A) the set up equipped with ReactIR probe, N_2 flow, pH meter (from left to right) B) validation of the ReactIR set up with MEA solution (1 M, 50 mL). When the MEA solution was treated with CO_2 at t = 20 min, the absorbances were observed*

upon the formation of MEA carbamate, at wavenumber 1563 cm^{-1} (COO^- asymmetric stretching vibration), 1492 cm^{-1} (COO^- symmetric stretching vibration), 1324 cm^{-1} ($N–COO^-$ bond stretching vibration) as reported.[34] *The intensity of the three absorbances were recorded and normalized with consistency, showing the same trends of MEA carbamate concentration profile on real-time. Panels C) and D): CO_2 capture and desorption experiments were recorded in the presence of PcAmd (4 μM) or same amount of enzyme-free storage buffer, respectively, in MEA solution (0.5 M in Milli-Q water, 25 mL). The spectrum of the solution was recorded (black curve) in comparison with the spectra of MEA-H_2O-CO_2 system. The peak at 1324 cm^{-1} (ν ($N–COO^-$)) became increasingly intensive while CO_2 bubbling, indicating the formation of MEA carbamate. CO_2 bubbling was stopped at pH 8.7 when enzyme or enzyme-free phosphate buffer was added to the mixture (red curve, bold). Afterwards, the N_2 purging gas (0.08 L/min) was on and the desorption experiments were recorded for another 45 min with an ending point pH ca. 9.4.*

5.5.1 (Bi)Carbonate accumulation accelerated under PcAmd-catalyzed conditions

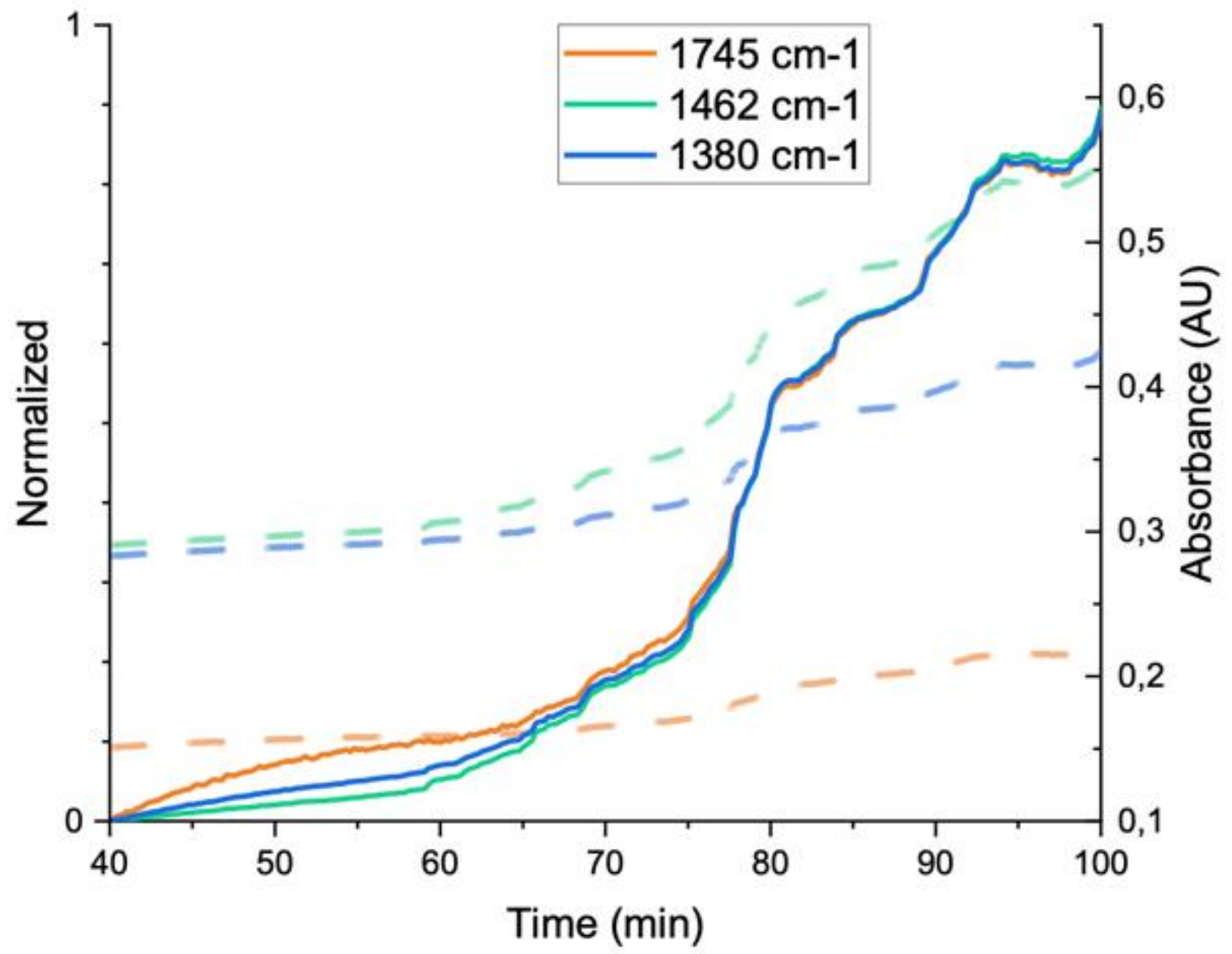

Figure S12. *PcAmd (4 uM) was added to the solution at t = 58 min, and the desorption experiment was monitored by in situ IR for another 45 min. The intensity of the three absorption bands at 1745, 1462, and 1380 cm^{-1} were correlated and assigned to carbonate species based on literature values.*[34] *The shoulder peak at 1364 cm^{-1} was assigned to bicarbonate*[33, 34, 35] *Based on literature studies, straightforward analysis of dissolved bicarbonate and carbonate*

is not feasible due to the overlap of both vibrations stretch and require curve deconvolution. [57] *However, the presence of carbonate and bicarbonate ions is evident at high pH over 9 according to Bjerrum plot. Therefore, we attributed the peak intensity to the formation of (bi)carbonate instead of other dissolved inorganic carbon species. We cannot quantify how much fractions of bicarbonate and carbonate peak contribute to these peaks, although our conclusion derived will be the same.*

5.5.2 Carbonic anhydrase showed limited effect on carbamate hydrolysis compared to Amidase and the control experiment without enzyme

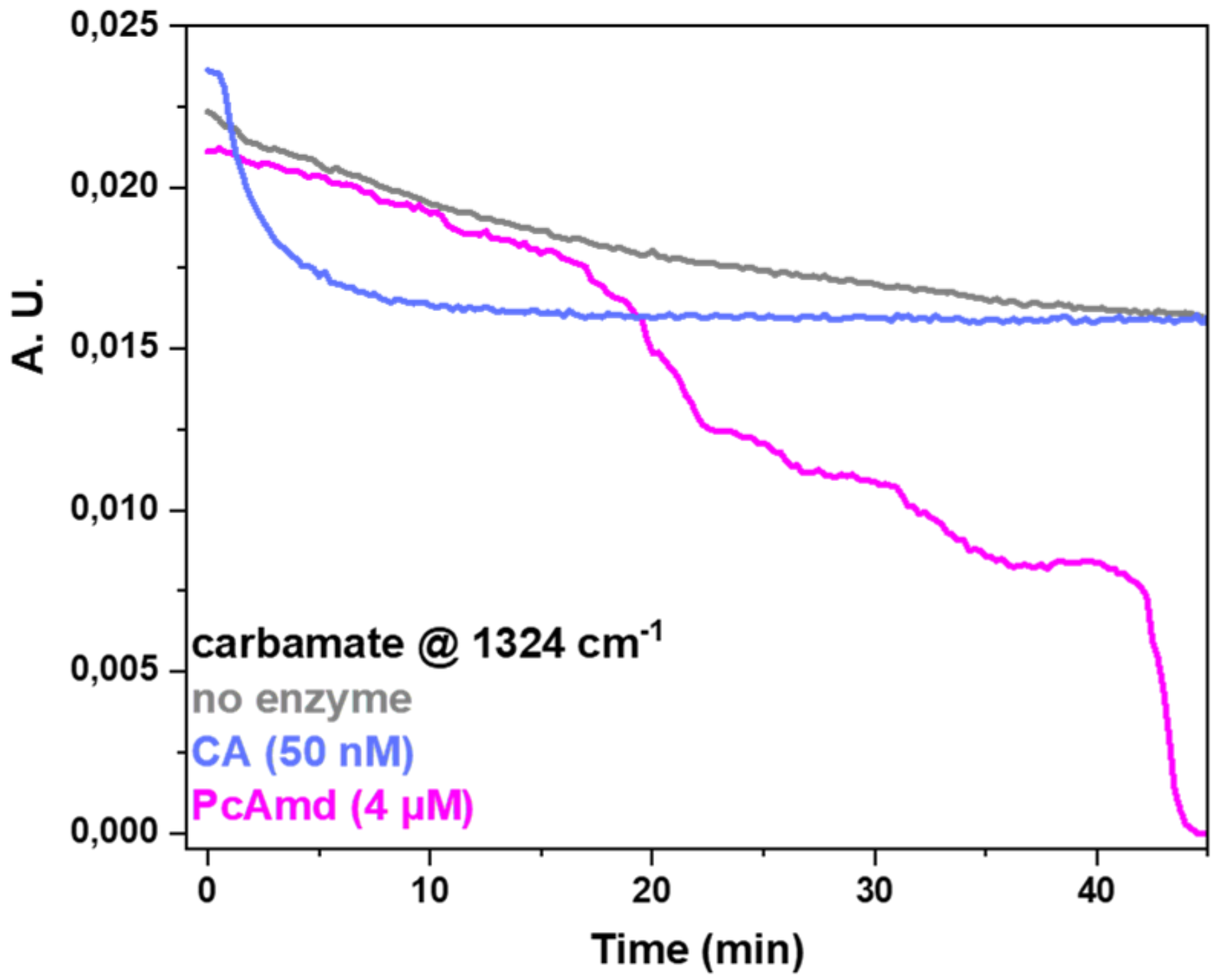

Figure S13. *CA promoted CO_2 desorption experiments were also monitored by in situ IR for comparison. Although the carbamate absorption band decreased very fast for the first 10 min (blue curve), it ended at the same ending point as the non-enzymatic control experiment (grey curve). This apparent activity can be explained by the high efficacy of CA in catalyzing bicarbonate dissociation, which shortened the time required for the equilibria to build up between bicarbonate and carbamate, compared to the non-catalytic experiment. In other words, CA can't directly catalyze carbamate hydrolysis, whereas PcAmd can (pink curve).*

6. Protons consumed for one molecule of CO_2 desorption from MEA solutions at pH 9.0

The following section describes how addition of protons from the pH-stat meaurements is converted to CO_2 desorption.

When CO_2 is bubbled into a MEA solution (for example, 20 mM) carbamate, bicarbonate, and carbonate are formed and an equilibrium is reached between the different components. See Scheme S1 below. The MEA-CO_2-H_2O system is very complex, and it has been described in detail elsewhere (refs. 13 and 14 in the main). The purpose of this section is to give a rough calculation of how many protons react to release one molecule of CO_2 at pH 9.0. Therefore, for simplicity a couple of approximations have been made. First, we ignore the carbonate species at pH 9.0, which corresponds to 5% according to the Bjerrum equation. Second, we ignore the effect of adding Cl^- ions to the solution when titrating with HCl in the pH-stat experiments, although the ionic strength might affect the p*K*a of MEA.[58]

To estimate how many protons are consumed for 1 molecule of CO_2 desorption from the MEA-CO_2-H_2O system at pH 9.0, quantifications of each species are necessary. The approximate concentrations are calculated in Scheme 1 using two different p*K*a values.

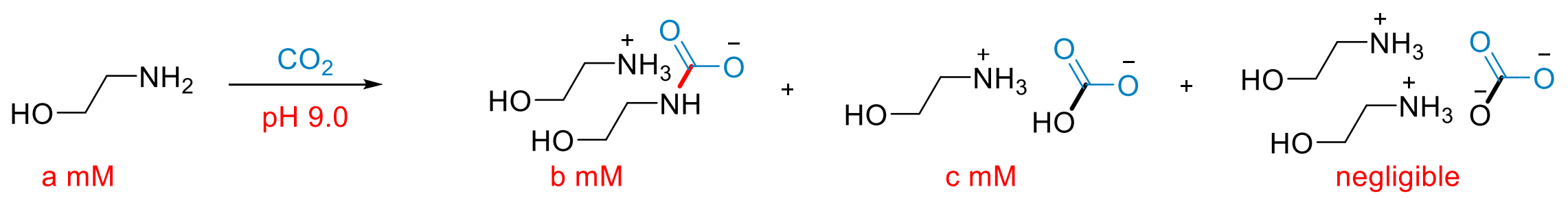

(equation 1) a + 2b + c = 20 mM

(equation 2) 46% = 2b / 20
experimental value by ^{1}H NMR

(equation 3) pH = p*K*a + log (a / (b+c))

⟹

$MEAH^+$	p*K*a 9.5	p*K*a 9.9
	a = 3.7	a = 1.7
	b = 4,6	b = 4,6
	c = 7.1	c = 9.1

Scheme S1. *The concentration of MEA carbamate (b mM) was determined from experimental values from ^{1}H NMR experiments. The concentrations of (a mM) and (c mM) were determined by equation 3 and these values depend on the pKa of protonated MEA. Here we used two different values of protonated MEA; pKa of 9.5, which is a typical litterature value, and pKa of 9.9, which was estimated experimentally. Approximate concentrations of neutral MEA (a mM), carbamate (b mM) and bicarbonate (c mM) species were calculated using both pKa values.*

With the use of the quantified values from Scheme S1 the average consumption of protons during CO_2 desorption can now be estimated, see Scheme S2. Since the carbonate species was considered negligible, the calculations only take carbamate and bicarbonate into consideration. Carbamate anion released 1 molecule of CO_2 and 1 neutral molecule of MEA, which needs to be protonated further to keep pH 9.0 unchanged. These amounts of protons were dependent again on the p*K*a of the protonated MEA.

In further detail, we can say that 1 carbamate anion consumed 1.76 or 1.89 protons given the p*K*a values of 9.5 or 9.9, respectively. In case of bicarbonate, 1 proton was consumed per bicarbonate molecule. The amounts of protons required for CO_2 desorption from carbamate and bicarbonate were calculated to be (1.76 * b * V) (or 1.89 * b * V) mmol and (1 * c * V) mmol, respectively.

Altogether, this means that on average 1.3 protons are consumed for 1 molecule of CO_2 desorption from MEA sorbent at a constant pH of 9.0. This value seems to be the same for both p*K*a values of the protonated MEA, indicating that variations in p*K*a have only minor effect on our rough calculations.

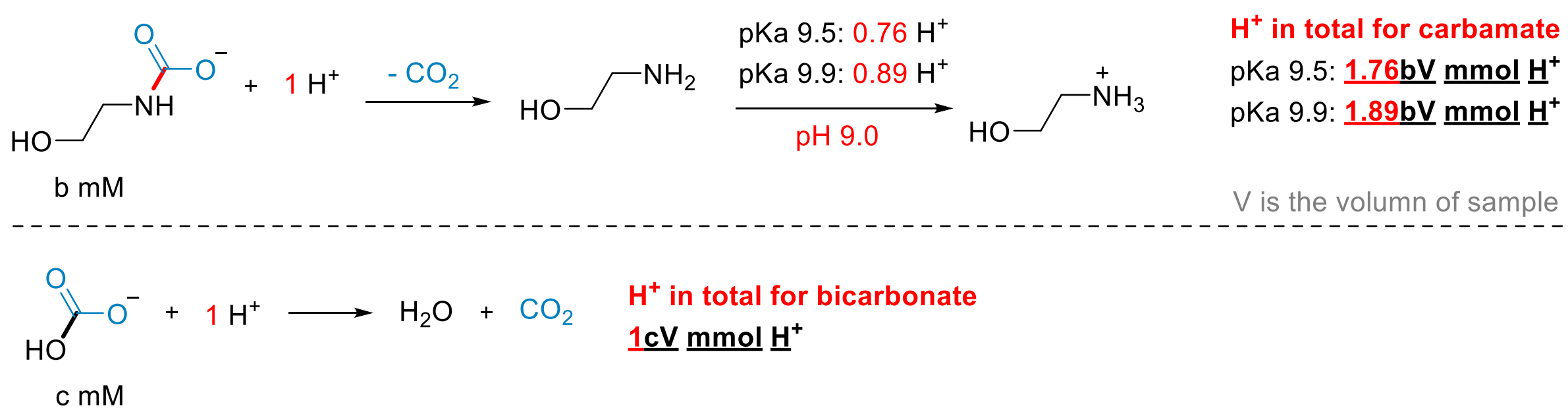

pKa of $MEAH^+$	H^+ in total (mmol)	CO_2 in total (mmol)	Average H^+/CO_2
9.5	1.76 bV + 1 cV	bV + cV	1.3
9.9	1.89 bV + 1 cV	bV + cV	1.3

Scheme S2. *Approximately 1.3 protons on average were required for 1 molecule of CO_2 desorption from the sorbent using two different pKa values of protonated MEA.*

References

[52] S. F. Altschul, W. Gish, W. Miller, E. W. Myers, D. J. Lipman, “Basic local alignment search tool” *J. Mol. Biol.* **1990**, *215*, 403–410.

[53] I. Letunic, P. Bork, “Interactive Tree of Life (iTOL) v6: Recent updates to the phylogenetic tree display and annotation tool” *Nucleic Acids Res.* **2024**, *52*, W78–W82.

[54] Neurosnap Inc. (2022). Neurosnap: An online platform for computational biology and chemistry. Available at: https://neurosnap.ai/

[55] S. Passaro, G. Corso, J. Wohlwend, M. Reveiz, S. Thaler, V. R. Somnath, N. Getz, T. Portnoi, J. Roy, H. Stark, D. Kwabi-Addo, D. Beaini, T. Jaakkola, R. Barzilay, “Boltz-2: Towards Accurate and Efficient Binding Affinity Prediction” **2025**, DOI: 10.1101/2025.06.14.659707.

[56] G. Corso, H. Stärk, B. Jing, R. Barzilay, T. Jaakkola, “DiffDock: Diffusion Steps, Twists, and Turns for Molecular Docking” **2023**. Doi: org/10.48550/arXiv.2210.01776.[57] M. Falk, A. G. Miller, “Infrared spectrum of carbon dioxide in aqueous solution” *Vib. Spectrosc.* **1992**, *4*, 105–108.

[58] A. Tagiuri, M. Mohamedali, A. Henni, “Dissociation Constant (p*K*a) and Thermodynamic Properties of Some Tertiary and Cyclic Amines from (298 to 333) K” *J. Chem. Eng. Data.* **2016**, *61*, 247–254.
